# An Integrated Gut Microbiota and Network Pharmacology Study on Fuzi-Lizhong Pill for Treating Diarrhea-Predominant Irritable Bowel Syndrome

**DOI:** 10.3389/fphar.2021.746923

**Published:** 2021-11-30

**Authors:** Zhang Zhen, Lin Xia, Huang You, Zhou Jingwei, Yang Shasha, Wei Xinyi, Lai Wenjing, Zhang Xin, Fu Chaomei

**Affiliations:** ^1^ State Key Laboratory of Southwestern Chinese Medicine Resources, Pharmacy College, Chengdu University of Traditional Chinese Medicine, Chengdu, China; ^2^ Key Laboratory of Quality Control and Efficacy Evaluation of Traditional Chinese Medicine Formula Granules, Sichuan New Green Medicine Science and Technology Development Co., Ltd., Pengzhou, China

**Keywords:** irritable bowel syndrome, Fuzi-Lizhong pill, gut microbiota, network pharmacology, crucial targets

## Abstract

Diarrhea-predominant irritable bowel syndrome (IBS-D) is one of the most common chronic functional gastrointestinal diseases with limited treatments. Gut microbiota play an important role in chronic gastrointestinal diseases. In traditional Chinese medicine (TCM), Spleen–Yang deficiency (SYD) is one of the root causes of IBS-D. Fuzi-Lizhong pill (FLZP) is well known for its powerful capacity for treating SYD and has a good clinical effect on IBS-D. However, the mechanism of FLZP on the gut microbiota of IBS-D has not been fully clarified. Our present study aimed to reveal the mechanism of FLZP regulating gut microbiota of IBS-D. The body mass, CCK, MTL, and Bristol fecal character score were used to verify the establishment of the IBS-D model. IL-6, TNF, IL-1β, and IFN-γ were crucial targets screened by network pharmacology and preliminarily verified by ELISA. Eighteen gut microbiota were important for the treatment of IBS-D with FLZP. Bacteroidetes, *Blautia*, *Turicibacter*, and *Ruminococcus_torques_group* were the crucial gut microbiota that FLZP inhibits persistent systemic inflammation in the IBS-D model*. Lactobacillus* is the crucial gut microbiota that FLZP renovates intestinal immune barrier in the IBS-D model. In summary, FLZP can affect bacterial diversity and community structures in the host and regulate inflammation and immune system to treat IBS-D.

## Introduction

Irritable bowel syndrome (IBS) is one of the most common functional gastrointestinal diseases with no organic pathological changes that can explain its symptoms, and its incidence is about 10–20% worldwide (Rusu and Dumitrascu, 2015). Diarrhea-predominant irritable bowel syndrome (IBS-D) is one of the most important phenotypes of IBS, with recurrent abdominal pain and diarrhea as the main clinical manifestations (Li et al., 2020). At present, most scholars believe that IBS-D is the result of multiple factors. Traditional treatments (such as antibiotics, gastrointestinal motion–regulating agents, and antipurgative agents) often target a specific mechanism (Lacy et al., 2021). They do not treat all the symptoms or root causes and can cause some side effects. Overuse of these drugs may derive new iatrogenic diseases (Nee et al., 2015). In recent years, studies have reported that traditional Chinese medicine (TCM) has achieved good results in treating IBS-D (Su et al., 2013; Li et al., 2018; Pei et al., 2018; Hou et al., 2019). In the theory of TCM, the disease belongs to the “Xiexie.” Spleen–Yang deficiency (SYD) is one of the root causes of IBS-D ( Zhang et al., 2017a ). If the spleen Yang is deficient, it will manifest as “not transporting,” which means the gastrointestinal movement would be disturbed, and “Xiexie” will occur ( Zhang et al., 2021a; Zhang et al., 2021b ). Different from the spleen in modern anatomy, “spleen” in TCM is a comprehensive concept of structures as well as functions: it is a composite structure mainly involving the spleen, pancreas, and lymphoid. Moreover, it involves multiple functions in the body such as digestive and immunologic function ( Li et al., 2003 ). The spleen is the hub for digesting food, distribution of cereal essence, and body fluid metabolism, and it plays a vital role in maintaining the basic functions of the human body ( Zhang et al., 2020 ). To clarify the root causes of IBS-D in TCM is conducive to the selection of appropriate Chinese medicine.

Fuzi-Lizhong pill (FLZP), a classic TCM prescription for SYD treatment, is originated in Taiping Huimin Heji Ju Fang in Song Dynasty (the year 1,102 by the Western calendar) (Zhao et al., 2013; Gan et al., 2017). It is a national essential drug and sold as OTC in China and used in the treatment of digestive system diseases for thousands of years, including enteritis, diarrhea, and gastritis (Hao and Qian, 2017; Yang, 2018; Wu, 2020). FLZP includes *Aconitum carmichaelii* Debx. (Fuzi), *Zingiber officinale* Rosc. (Ganjiang), *Glycyrrhiza uralensis* Fisch. (Gancao), *Codonopsis pilosula* (Franch.) Nannf. (Dangshen), and *Atractylodes macrocephala* Koidz. (Baizhu) (Jiang et al., 2019). It is well known that Fuzi is a poisonous Chinese herb. In the TCM theory, the toxicity of Fuzi can be eliminated by processing it or decocting it for a long time (Wei et al., 2019). Besides, the combination of licorice can reduce the toxicity of Fuzi (Zhang et al., 2012; Ma et al., 2019). Moreover, the form of traditional Chinese medicine pill can also be used to reduce toxicity in ancient China, and TCM pills are similar to modern sustained-release preparations (Zhang et al., 2017b). At the same time, the *Chinese Pharmacopoeia* published over the years also stipulated that toxic aconitine components in FLZP should be strictly controlled. Researchers also used RRLC-MS-MS to detect aconitine components in FLZP so as to improve the quality control standard (Dong et al., 2018). FLZP has also been proven to be effective in treating IBS-D in modern clinical applications (Ye, 2010; Zheng et al., 2019; Meng and Guo, 2019). However, its specific mechanism is not completely clear.

In recent years, gut microbiota have been found to be closely related to the occurrence of gastrointestinal diseases (Zuo and Ng, 2018; Cani and Jordan, 2018). A large number of studies have also shown that gut microbiota disorder plays an important role in the pathogenesis of IBS-D (Collins, 2014; Li et al., 2018; Zhuang et al., 2018). Meanwhile, network pharmacology can provide insights into the molecular mechanisms of herbal prescriptions at the molecular and systematic levels (Zhang et al., 2017c). Recently, some researchers have successfully used the integrated network pharmacology and gut microbiota analysis strategy to explore the interactions between organisms and TCM prescriptions, bringing great inspiration to the mechanism research of FLZP (Gao et al., 2018; Meng et al., 2020). In this study, network pharmacology and gut microbiota analysis were applied to explore the effects of FLZP regulating gut microbiota of IBS-D, which could provide a basis for the clinical application of FLZP treating IBS-D.

## Materials and Methods

### Preparation of Administration

*Aconitum carmichaelii* Debx. (No. 1804038), *Zingiber officinale* Rosc. (No. 1806012), *Glycyrrhiza uralensis* Fisch. ex DC. (No. 1807031), *Codonopsis pilosula* (Franch.) Nannf. (No. 1806128), *Atractylodes macrocephala* Koidz. (No. 1803009), and *Cassia angustifolia* Vahl (No. 1711080) were purchased from Sichuan Neautus Traditional Chinese Medicine Co., Ltd. (Chengdu, China) and were authenticated by Prof. Jin Pei, Department of Pharmacognosy of Chengdu University of Chinese Medicine. All five herbs were grounded into fine powders and weighed according to the instructions recorded in *Chinese Pharmacopoeia* (2015 edition) and then mixed well (Chinese Pharmacopoeia Commission, 2015). Honey was heated at 116°C∼118°C until bright yellow uniform bubbles appeared on the surface and the honey became sticky. Mixed power and thermal refined honey were mixed at a ratio of 1:0.8 and were made into FLZP (there was 0.153 kg of crude aconite for every 1 kg of FLZP). The chemical characterization analysis of FLZP was shown in Supplementary Material by HPLC-Q-TOF-MS (Supplementary Table S1). Meanwhile, the quality control method of FLZP was established according to *Chinese Pharmacopoeia*. Moreover, a method for the determination of major compounds in FLZP was developed. The content of liquiritin was 2.916 mg/g, glycyrrhizin was 5.617 mg/g, benzoylmesaconine was 0.426 mg/g, benzoylaconine was 0.030 mg/g, and benzoylhypaconine was 0.024 mg/g. The quantitative analysis of these five compounds including HPLC-MS conditions (Supplementary Table S2), the standard curve equations (Supplementary Table S3), and sample preparation and chromatograms (Supplementary Figure S2 and Supplementary Figure S3) are shown in Supplementary Material.

Senna water extract: *Cassia angustifolia* Vahl was placed in a constant temperature deionized water bath at 70°C overnight; after filtering, each 100 ml contained 100 g of crude drug, and then the filtrate was stored in a refrigerator at 4°C for later (Wu and Huang, 2015).

### Animals and Drug Administration

Sprague-Dawley (SD) male rats (250 ± 20 g) and their food were purchased from Sichuan DaShuo Biotechnology Co., Ltd. (Chengdu, China) (Certificate No. SCXK (Chuan) 2015-030). Before the experiment, all of the rats were housed in an animal room with a controlled environment (20–25°C, 65–69% relative humidity, 12 h dark–light cycle) and were given water and fed normal food. All of the experiments and procedures were performed according to the Regulations of Experimental Animal Administration issued by the State Committee of Science and Technology of China (2017), and the protocol was approved by the Committee on the Ethics of Animal Experiments of Chengdu University of Traditional Chinese Medicine (No. 2018-15).

After 1 week for acclimatization, 10 SD rats were randomly assigned to group A: the normal group, which was only given water and fed normal food throughout the study. The other rats were used to establish the IBS-D model in the state of SYD by using the compound factor modeling method with the principle of “indiscipline in diet + excessive fatigue stimulus + intragastric administration of senna water extract” (Luo et al., 2019; Zeng et al., 2019; Li et al., 2020). The detailed operational procedures were as follows: for the first 6 days, each rat was fed 10 g cabbage on odd days and 10 g high fat mixed feed on even days. Additionally, the rats were made to swim until fatigued every afternoon (water temperature: 38°C) (the standard of fatigue was determined by the phenomenon that the entire neck submerged under the water and the inability to continue swimming). From 7 to 21 days, the operation of the previous 6 days was maintained and combined with intragastric administration of senna water extract (10 ml/kg). The following characteristics appeared in the model rats that indicated the SYD model was made successfully: loose stool, significant weight loss, gathering together for warming, loss of appetite, withered and dull hair, significantly reduced swimming time, and mucous around the anus (Ning et al., 2017). The body mass, CCK, MTL, and Bristol fecal character score were used to judge the success of the IBS-D model (Liang et al., 2004; Liang et al., 2012; Li et al., 2020; Scuderi et al., 2020). The fecal traits were characterized using the Bristol stool typing score. In brief, standard for the Bristol stool typing score is shown in Supplementary Table S4.

After successful modeling, 30 IBS-D model rats were randomly divided into three groups: group B (model group), group C (low-dosage group), and group D (high-dosage group). FLZP was dissolved in 0.5% carboxymethyl cellulose sodium (CMC-NA) (Chengdu Gulong Chemical Co., Ltd., Chengdu, China) and ground to prepare FLZP low-dosage suspension (50 mg crude drug/ml) and FLZP high-dosage suspension (150 mg crude drug/ml). During days 22–50, the FLZP low-dose suspension was chosen in the present study for rats of group C, and the FLZP high-dose suspension was chosen for rats of group D. Group B was given an equal amount of normal saline every day. Equivalent conversion is based on the surface area of human body and animal body. Groups B, C, and D were continuously made into an IBS-D model.

### Sample Collection and Preparation

All the rats were prohibited any food for 12 h before the last administration. On the 51st day, serum samples were collected from the abdominal aorta of all the rats after 45 min of intragastric administration. The serum samples were left out at room temperature for 1 h and were centrifuged at 3,000 rpm for 15 min at 4°C, and then the supernatant was transferred to frozen pipes. Fresh stool samples were quickly collected in frozen pipes. All the biological samples had been preserved at −80°C until the assays were performed.

### Network Pharmacology Study

#### Active Ingredients and Therapeutic Target Database Building

All of the chemical ingredients of FLZP were obtained from Traditional Chinese Medicine Systems Pharmacology Database and Analysis Platform (TCMSP, http://lsp.nwu.edu.cn/tcmsp.php), and active ingredients were selected using the *in silico* integrative ADME model. The ADME system used in this study included prediction of oral bioavailability (OB) and drug-likeness (DL), and ingredients were retained only if OB ≥ 30 and DL ≥ 0.18 to satisfy criteria suggested by the TCMSP database. The reason why we selected this rule was that molecules with OB ≥ 30% was considered to possess the high oral absorption and utilization, and the average index of molecules with biological properties was 0.18 in the DrugBank database (Li et al., 2019).

Target genes of active ingredients in FLZP were screened and predicted by databases of TCMSP and SwissTarget Prediction (http://www.swisstargetprediction.ch/) database. Then these targets were normalized by UniProt databases successively. Subsequently, IBS-D–causing target genes were identified through the GeneCards database (https://www.genecards.org/) and OMIM database (https://omim.org/). Target genes of active ingredients in FLZP were matched with target genes of IBS-D. Then the therapeutic targets of FLZP treating IBS-D were harvested.

### Pathway Analysis of Therapeutic Targets

In order to investigate the biological effects of therapeutic targets on IBS-D through regulating specific pathways, KEGG (Kyoto Encyclopedia of Genes and Genomes) analysis of therapeutic targets was screened using DAVID 6.7 (FDR<0.05).

### Network Construction and Analysis

Network construction was visualized using Cytoscape 3.7.0 as follows: 1) protein–protein interaction (PPI) network and the therapeutic targets in PPI of FLZP in treating IBS-D were constructed by STRING database and Cytoscape, in which the network targets were screened with high confidence (0.7). 2) Component–target (C-T) network, in order to explore kernel ingredients of FLZP in treating IBS-D as well as to disclose the synergistic effects of multicomponent and multitarget in FLZP, C-T network was established based on the binding of active compounds to the corresponding targets.

### ELISA

In order to verify the establishment of the IBS-D model and the crucial targets in FLZP in treating IBS-D, the levels of cholecystokinin (CCK) (Cusabio, Wuhan, China), motilin (MTL) (Elabcience Biotechnology Co., Ltd., Hangzhou, China), interleukin-6 (IL-6) (Multisciences Biotech, Co., Ltd., Hangzhou, China), tumor necrosis factor-alpha (TNF-α) (Multisciences Biotech, Co., Ltd., Hangzhou, China), interleukin-1beta (IL-1β) (Multisciences Biotech, Co., Ltd., Hangzhou, China), and interferon-gamma (IFN-γ) (Multisciences Biotech, Co., Ltd., Hangzhou, China) were detected from serum samples collected after the end of experimental administration by using ELISA kits, according to the manufacturers’ instructions.

### Gut Microbiota Study

#### DNA Extraction and PCR Amplification

Total bacterial DNA was extracted from fresh stool samples by HiPure Soil DNA Kit B (Axygen Biosciences, Union City, CA, United States) according to the manufacturer’s instructions. The quantity and quality of the extracted DNA were measured using Qubit 2.0 Fluorometer (Invitrogen, Carlsbad, CA). The V3–V4 region of 16S rRNA was amplified by polymerase chain reaction (PCR) with the forward primer (CCTACGGRRBGCASCAGKVRGAAT) and reverse primer (GGACTACNVGGGTWTCTAATCC). The amplification system was prepared as follows: TransStart Buffer (2.5 μl), dNTPs (2 μl), primers (1 μl *2), TransStart Taq DNA (0.5 μl), DNA template (20 ng), and ddH2O (added to 25 μl). The amplification conditions were followed by 24 cycles of initial denaturation at 94°C for 3 min, consisting of denaturation at 94°C for 5 s, annealing at 57°C for 90 s, and extension at 72°C for 10 s, with a final extension at 72°C for 5 min. The resulting PCR products were measured using 0.8% agarose gel electrophoresis.

### High-Throughput Sequencing

Library concentrations were tested by Qubit 3.0 Fluorometer (Invitrogen, Carlsbad, CA) and quantified to 10 nM. PE250/FE300 double-end sequencing was performed using to Illumina MiSeq (Illumina, San Diego, CA, United States) and the sequence information was read by MiSeq Control Software (MCS).

### Bioinformatics Analysis

Operational taxonomic units (OTUs) were clustered with a 97% similarity cut-off using VSEARCH (version 1.9.6) after mass filtering and removal of chimeric sequences. The taxonomy of each 16S rRNA gene sequence was analyzed by the RDP Classifier algorithm against the Silva 132 16S rRNA database (http://www.arb-silva.de/). The rarefaction curve and species accumulation curve could be used to judge whether the sequencing depth of each sample and sample size was sufficient to reflect the microbial diversity in the community samples. The flatter the curve was, the more sufficient the sequencing resulted. Alpha diversity analysis, which included determination of the Chao1, ACE, Shannon, and Simpson indices, was used to evaluate abundance and diversity. Beta diversity analysis showed the natural distribution of samples which reflected the similarity between samples. LEfSe analysis was used to screen the biomarkers based on the linear discriminant analysis (LDA) effect size (LDA >3).

### Statistical Analysis

Statistical analysis was performed by using SPSS 19.0 software, and all data were expressed as mean ± SD. The statistical results were conducted with two-tailed unpaired Student’s t test. Correlation analysis was used by Spearman correlation. The value of *p* ≤ 0.05 was regarded significant difference, and the value of *p* ≤ 0.01 was regarded extremely significant difference.

## Results

### Establishment and Therapeutic Effect of IBS-D Model

**FIGURE 1:**
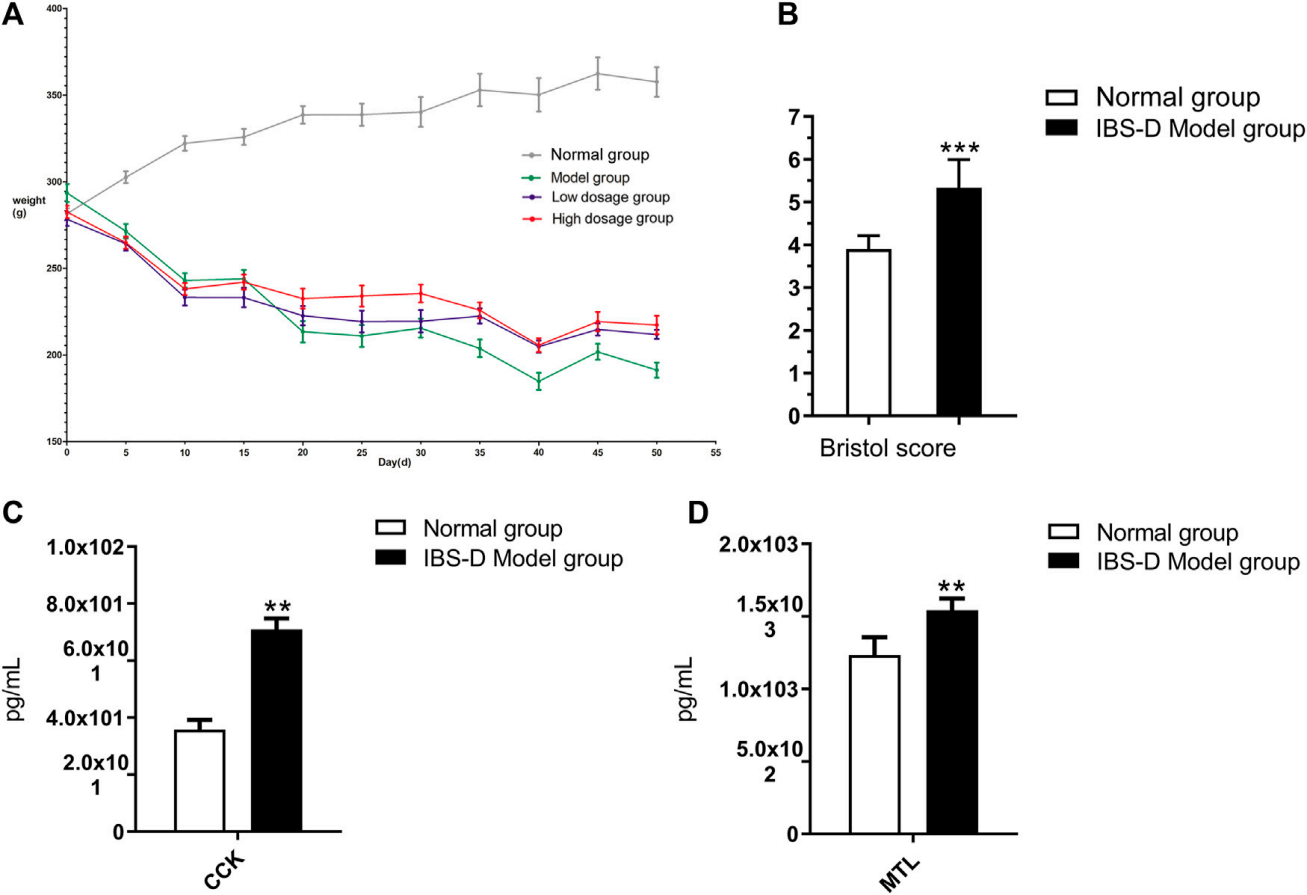
Body weight changes **(A)**, the Bristol fecal character score **(B)**, the level changes of CCK **(C)**, and MTL **(D)**. *p < 0.05 and **p < 0.01 vs. normal group. ^▲^ *p* < 0.05 and ^▲▲^ *p* < 0.01 vs. model group.

To explore the characteristics of the disease symptoms, the body mass, CCK, MTL, and Bristol fecal character score on the index were used. After 21 days of modeling, the body mass, CCK, MTL, Bristol fecal character score, and visceral index showed that there was significant difference between the results of the model rats and normal group (*p* < 0.05) (Figure 1). After administration, the above symptoms were significantly relieved in groups C and D compared with group B.

As shown in Figure 1A, compared with groups A, the weight of groups B, C, and D were obviously reduced, while the downward trend slowed significantly in groups C and D after the administration of FLZP. A statistical analysis of the mass data of each group on 21st day showed that there were significant differences between the normal group and groups B, C, and D (*p* < 0.01). In addition, a statistical analysis of the mass data of each group on 50th day showed that there were significant differences between the normal group and group B (*p* < 0.01), and there were also significant differences between group C and group D compared to group B (*p* < 0.01). As shown in Figure 1B, the Bristol fecal character score in the model group was significantly increased than that in the normal group (*p* < 0.01). The Bristol fecal character score were all decreased after FLZP treatment. Group D had statistically significant (*p* < 0.01). As shown in Figure 1C and Figure 1D, CKK and MTL in the model group were significantly increased than that in the normal group (*p* < 0.01). CKK and MTL were significantly decreased after FLZP treatment (*p* < 0.05). The above results highlighted the therapeutic effect of FLZP on IBS-D in rats.

### Network Pharmacology Study

#### Potential Compounds and Therapeutic Targets of FLZP

According to the ADME model, 143 potential compounds (OB ≥ 30%, DL ≥ 0.18) of five herbal medicines in FLZP prescription were identified (Supplementary Table S5), including 20 compounds from *Aconitum carmichaelii* Debx. (Fuzi), 20 compounds from *Codonopsis pilosula* (Franch.) Nannf. (Dangshen), seven compounds from *Atractylodes macrocephala* Koidz. (Baizhu), 90 compounds from *Glycyrrhiza uralensis* Fisch. (Gancao), four compounds from *Zingiber officinale* Rosc. (Ganjiang), one compound from Dangshen and Gancao, and one compound from Fuzi, Gancao, and Ganjiang.

1,079 predictive targets of FLZP were identified by TCMSP and SwissTarget Prediction database, and 164 IBS-D–associated target genes were obtained from GeneCards and OMIM database. After combining and analyzing the targets of FLZP and IBS-D, 69 therapeutic targets were obtained.

### KEGG Analysis of Therapeutic Targets

To examine the signaling pathways and functions of therapeutic targets, we performed functional enrichment analysis using KEGG pathways. 77 pathways were screened by FDR <0.05, and these pathways are presented in Supplementary Table S6. These pathways were classified into two main categories, including diseases-related pathways and immune-related pathways. Diseases-related pathways mainly included pathways in cancer (hsa05200), inflammatory bowel disease (hsa05321), and hepatitis B (hsa05161). Immune-related pathways mainly included intestinal immune network for IgA production (hsa04672), TNF (hsa04668), NF-kappa B (hsa04064), and Toll-like receptor (hsa04620) signaling pathway.

### Crucial Targets Selection and Analysis

**FIGURE 2:**
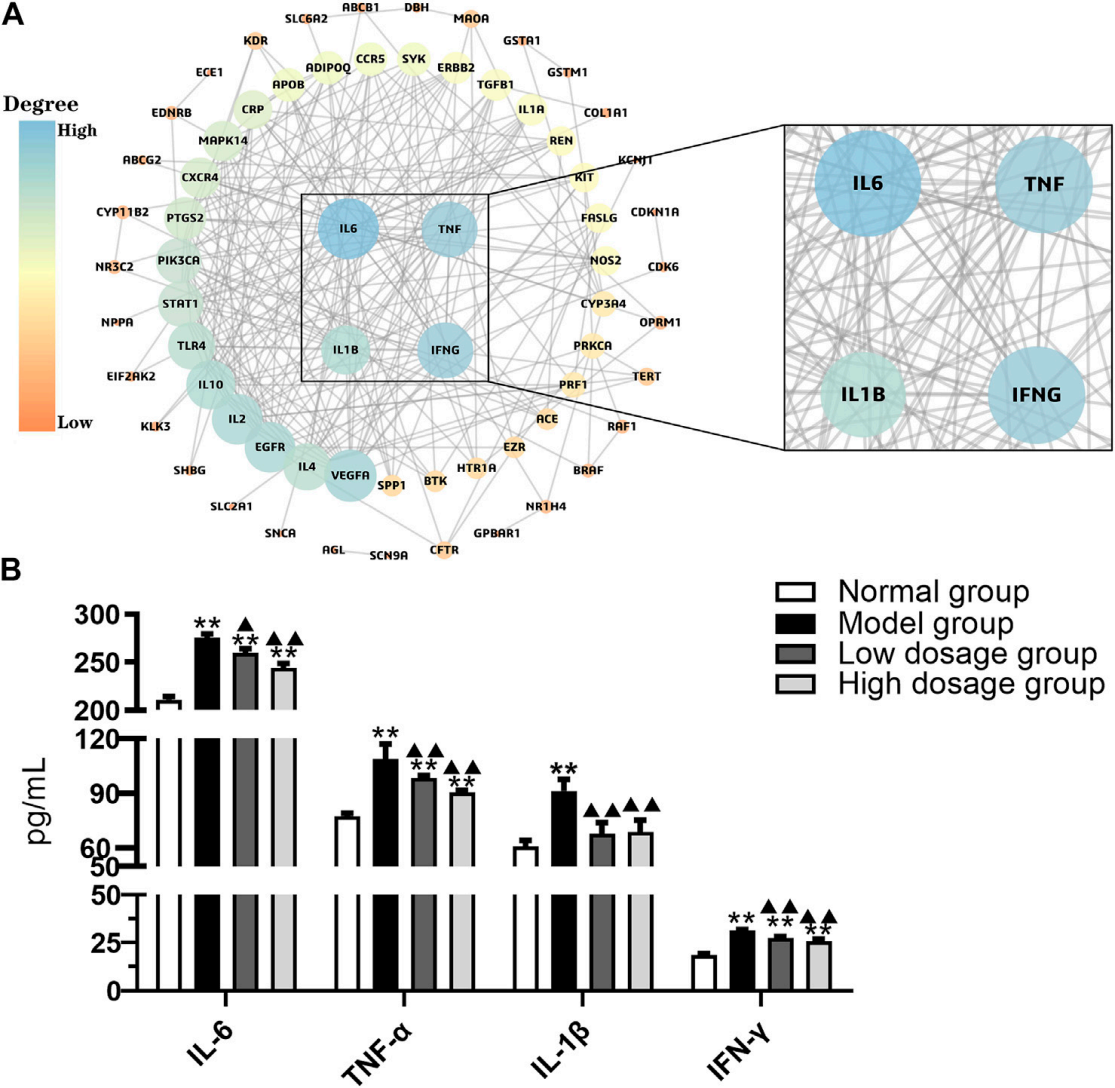
PPI network **(A)** and the level changes of IL-6, TNF-α, IL-1β, and IFN-γ **(B)**. *p < 0.05 and **p < 0.01 vs. normal group. ^▲^ *p* < 0.05 and ^▲▲^ *p* < 0.01 vs. model group.

The PPI network of therapeutic targets was constructed using Cytoscape software based on the STRING database. As a result, PPI network was constructed including 69 nodes and 240 edges (Figure 2A). IL6, TNF, IL-1β, and IFN-γ were identified as crucial targets with degree value more than 7.27 (the mean of degree). Then the four targets were validated using ELISA. As shown in Figure 2B, IL-6, TNF-α, IL-1β, and IFN-γ levels expression was significantly increased in the model group compared with the normal group (*p* < 0.01). It verified that crucial targets of immune regulation and anti-inflammatory have increased in the IBS-D model. Compared with the model group, IL-6, TNF-α, IL-1β, and IFN-γ levels expression in serum were significantly decreased in FLZP administration groups (*p* < 0.05). The above results revealed that FLZP can regulate these crucial targets for treating IBS-D.

### Kernel Ingredients Analysis

**FIGURE 3:**
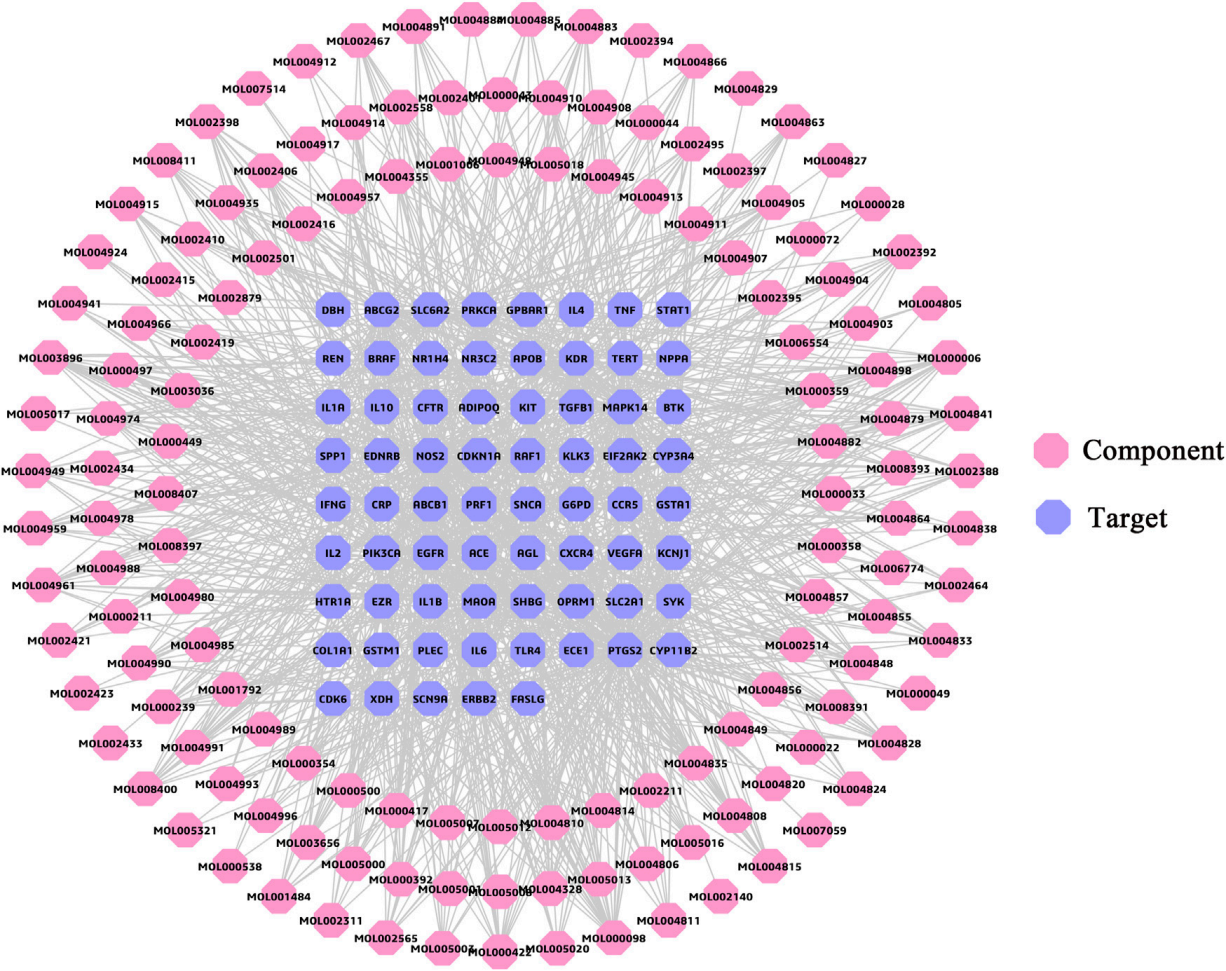
Component–target network. The pink regular octagon represents compound, and the lilac regular octagon represents the target.

The C-T network (Figure 3) consisted of 210 nodes (141 components, 69 targets) and 1,112 edges. The degree parameter of topological analysis was chosen to identify kernel ingredients. The result of network analysis showed that the average degree of compounds was 8.89. Meanwhile, degree of 45 compounds greater than the average value is presented in Supplementary Table S7, such as quercetin and luteolin, suggesting that these components might be kernel ingredients in FLZP in treating IBS-D. At the same time, these were also candidate components for leading compounds.

### Gut Microbiota Analysis

**FIGURE 4:**
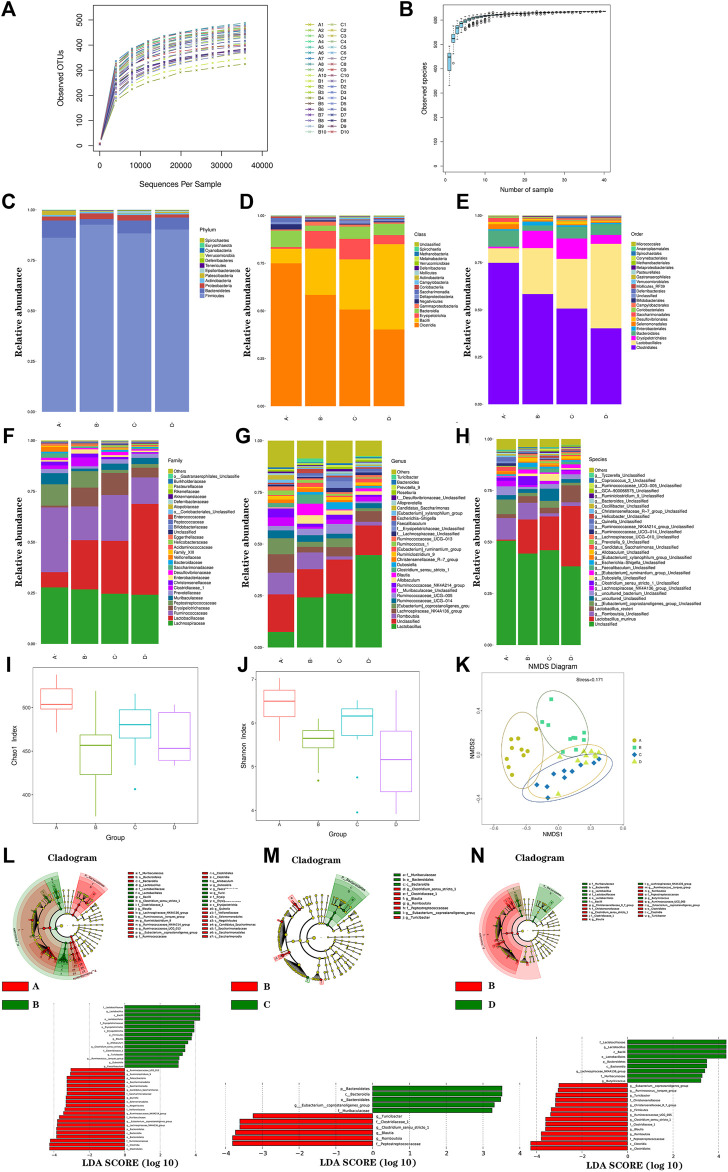
Gut microbiota analysis. The rarefaction curve **(A)**; species accumulation curve **(B)**; histogram of species distribution at different levels **(C)**; phylum; **(D)**, class; **(E)**, order; **(F)**, family; **(G)**, genus; **(H)**, species; Chao1 index **(I)**, Shannon index **(J)**, NMDS analysis **(K)**, LEfSE diagram of characteristic in gut microbiota between groups **(L–N)**. A, normal group; B, model group; C, low-dosage group; D, high-dosage group.

**FIGURE 5:**
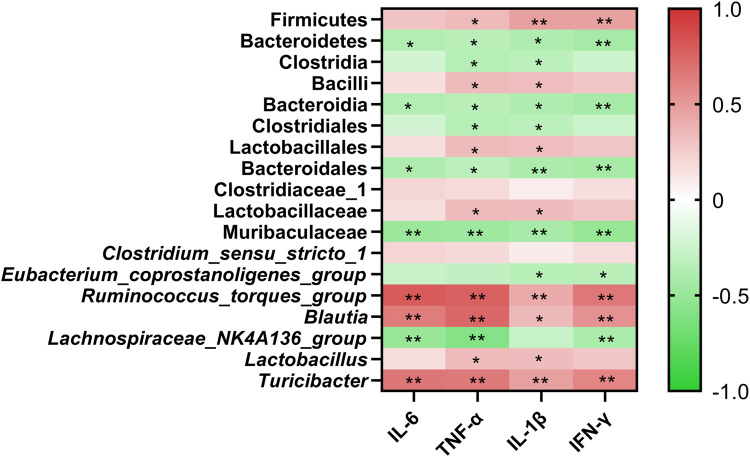
Clustering heatmap based on Spearman correlation. Line color represents the direction of correlation: red (positive), green (negative), the deeper the color, and the stronger the correlation. *p < 0.05, and **p < 0.01.

**FIGURE 6:**
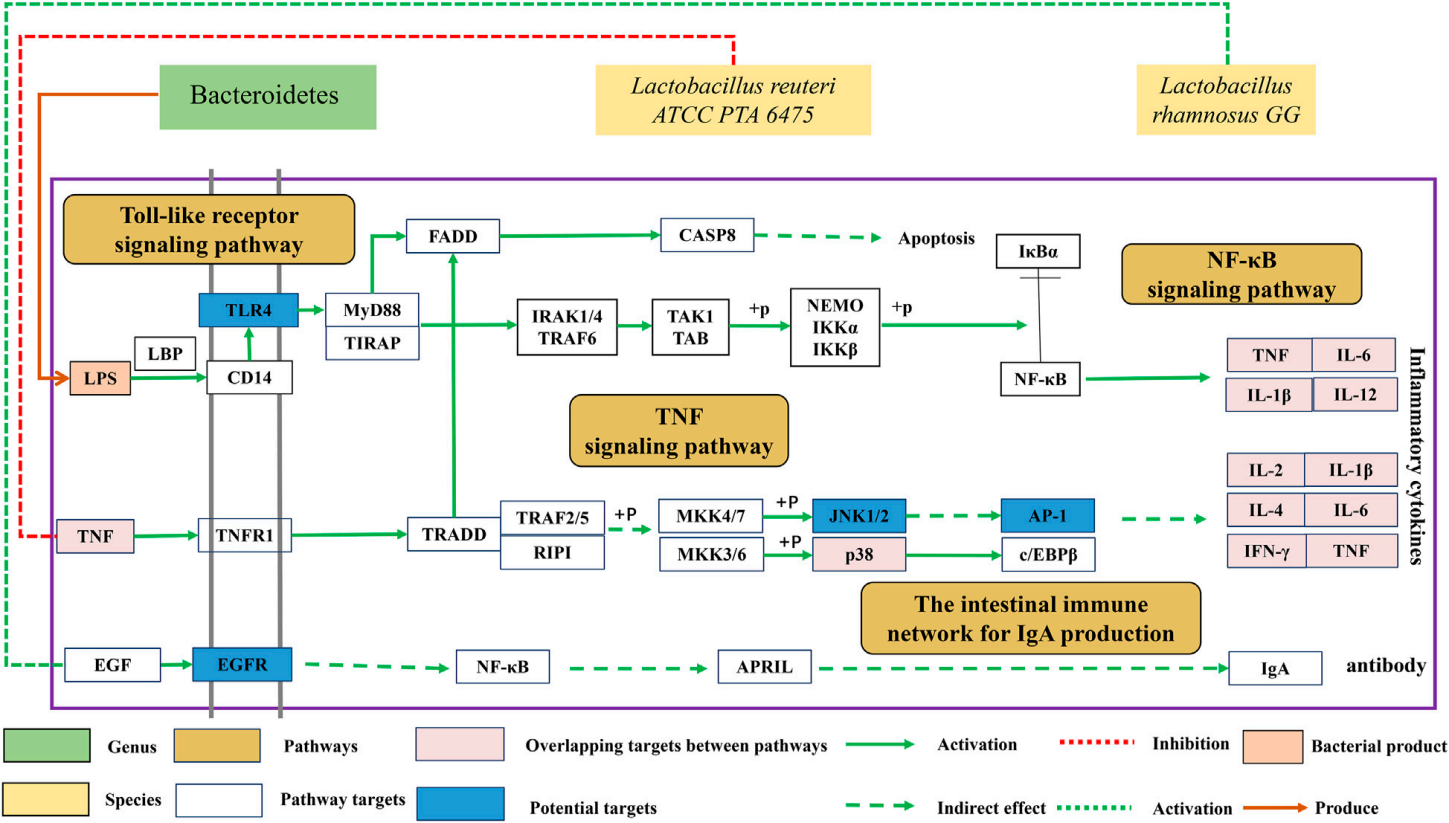
Mechanism of FLZP regulating gut microbiota of IBS-D.

In this study, a total of 2,423,687 high-quality reads were acquired from the 40 stool samples with an average of 60,592 sequences for each sample. After quality and chimera checking, 599 OTUs were determined (with 97% similarity). As shown in Figure 4A, the rarefaction curve revealed the sufficient sequencing depth in this study. As shown in Figure 4B, the species accumulation curve revealed the sufficient sample size in this study. Furthermore, bacterial community structures in different groups were presented at the phylum, class, order, family, genus, and species levels in Figures 4C–6H by using a taxonomic analysis.

The species abundance and diversity of every sample were represented by alpha diversity. The Chao1, Ace, Shannon, and Simpson indexes were the four common measurement indexes. As shown in Figure 4I and Figure 4J, the model group significantly reduced ACE (*p* < 0.01) and Chao1(*p* < 0.01) compared with the normal group. It suggested that microbial communities were reduced in the model group. Meanwhile, microbial communities showed increased trend in FLZP administration groups. Besides, the level of Shannon (*p* < 0.01) and Simpson (*p* < 0.01) were lower in the model group, which indicated the fewer diverse microbial communities. In general, model rats had significant structural changes of gut microbiota. To further analyze overall structural changes of the gut microbiota among different groups, NMDS diagram based on the Bray–Curtis distance was performed as shown in Figure 4K. The farther the distance between sample points in Figure 4K, the lower the similarity of samples was. The points in a group were concentrated. Evident separation of gut microbiota was observed among different groups. The bacterial members showed high differences in the model group compared with the normal group. FLZP administration groups showed high similarities in bacterial members with the normal group. The results revealed that FLZP had a significant restorative effect on the disorderly gut microbiota in the IBS-D model.

To explore the crucial gut microbiota in IBS-D and in FLZP in treating IBS-D, a Lefse difference analysis of taxon abundance was applied in this study. As shown in Figures 4L–N, 6, in the model group, the relative abundances of Firmicutes, Bacilli, Erysipelotrichia, Lactobacillales, Erysipelotrichales, Lactobacillaceae, Clostridiaceae, Erysipelotrichaceae, *Lactobacillus*, *Clostridium_sensu_stricto_1*, *Blautia*, *Ruminococcus_torques_group*, *Allobaculum*, *Dubosiella*, *Faecalibaculum*, and *Turicibacter* were higher than these in the normal group. Meanwhile, the relative abundances of Bacteroidetes, Patescibacteria, Bacteroidia, Clostridia, Negativicutes, Saccharimonadia, Bacteroidales, Clostridiales, Selenomonadales, Saccharimonadales, Muribaculaceae, Ruminococcaceae, Veillonellaceae, Saccharimonadaceae, Lachnospiraceae*_NK4A136_group*, *Ruminiclostridium_9*, Ruminococcaceae*_NK—4A214_group*, Ruminococcaceae*_UCG_013*, *Eubacterium_coprostanoligenes_group*, *Quinella*, and *Candidatus_Saccharimonas* were lower than these in the normal group. These thirty-seven gut microbiota can be regarded as the biomakers of IBS-D. After FLZP administration, significant changes were found in eighteen gut microbiota of IBS-D, which were regarded as the crucial gut microbiota. Specific results are shown in Table 1.

**TABLE 1:** Results of identification microflora analysis (*n* = 10).

Flora	LDA score B&A	Trend B&A	Trend C&B	Trend D&B
Phylum				
Firmicutes	3.800	↑	\	↓
Bacteroidetes	3.850	↓	↑	↑
Phylum-Class				
Firmicutes-Clostridia	4.242	↓	\	↓
Firmicutes-Bacilli	4.245	↑	\	↑
Bacteroidetes-Bacteroidia	3.850	↓	↑	↑
Phylum-Class-Order				
Firmicutes-Clostridia-Clostridiales	4.242	↓	\	↓
Firmicutes-Bacilli-Lactobacillales	4.245	↑	\	↑
Bacteroidetes-Bacteroidia-Bacteroidales	3.850	↓	↑	\
Phylum-Class-Order-Family				
Firmicutes-Clostridia-Clostridiales-Clostridiaceae_1	3.403	↑	↓	↓
Firmicutes-Bacilli-Lactobacillales-Lactobacillaceae	4.247	↑	\	↑
Bacteroidetes-Bacteroidia-Bacteroidales-Muribaculaceae	3.697	↓	↑	↑
Phylum-Class-Order-Family-Genus				
Firmicutes-Clostridia-Clostridiales-Clostridiaceae_1-*Clostridium_sensu_stricto_1*	3.406	↑	↓	↓
Firmicutes-Clostridia-Clostridiales-Eubacteriaceae-*Eubacterium__coprostanoligenes_group*	3.801	↓	↑	↓
Firmicutes-Clostridia-Clostridiales-Lachnospiraceae-*Ruminococcus__torques_group*	3.077	↑	\	↓
Firmicutes-Clostridia-Clostridiales-Lachnospiraceae-*Blautia*	3.767	↑	↓	↓
Firmicutes-Clostridia-Clostridiales-Lachnospiraceae-*Lachnospiraceae_NK4A136_group*	3.811	↓	\	↑
Firmicutes-Bacilli-Lactobacillales-Lactobacillaceae-*Lactobacillus*	4.247	↑	\	↑
Firmicutes-Erysipelotrichia-Erysipelotrichales-Erysipelotrichaceae-*Turicibacter*	3.264	↑	↓	↓

↑: The microflora is upregulated. ↓: The microflora is downregulated.

### Correlation Between Crucial Gut Microbiota and Crucial Targets

To better understand the functional correlation between crucial gut microbiota alterations and crucial targets, the Spearman correlation analysis was performed. As shown in Figure 5, at the phylum level, Firmicutes was positively correlated with TNF-α, IL-1β, and IFN-γ. Bacteroidetes was negatively correlated with IL-6, TNF-α, IL-1β, and IFN-γ. At the genus level, *Ruminococcus_torques_group*, *Blautia*, and *Turicibacter* were positively correlated with IL-6, TNF-α, IL-1β, and IFN-γ. *Lactobacillus* was positively correlated with TNF-α and IL-1β. Lachnospiraceae*_NK4A136_group w*as negatively correlated with IL-6, TNF-α, and IFN-γ. *Eubacterium_coprostanoligenes_group* was negatively correlated with IL-1β and IFN-γ.

## Discussions

As a crucial mutualistic part of the body, like an organ, gut microbiota can influence the host’s disease and health status through the modulation of signal pathways (Bäckhed et al., 2005; Thomasa and Versalovic, 2010; Sommer et al., 2017). A significant correlation between gut microbiota disorder and IBS has been found in studies. The potential mechanism is that gut microbiota disorder causes persistent chronic inflammation and excessive immune response of intestinal mucosa, which eventually induces the occurrence of IBS (Collins, 2014). In this study, FLZP could improve the gut microbiota structure to treat IBS-D. These regulated gut microbiota were regarded as the crucial gut microbiota, including Bacteroidetes, *Blautia*, *Turicibacter*, *Ruminococcus_torques_group*, and *Lactobacillus*. The main mechanisms may be as follows: one is to inhibit the proliferation of intestinal microflora and its products those can cause systemic inflammation. The second is to help the proliferation of probiotics those can promote the renovation of intestinal immune barrier.

### Inhibition of Persistent Systemic Inflammation

In this study, FLZP was found to regulate gut microbiota related to inflammation, including Bacteroidetes, *Blautia*, *Turicibacter*, and *Ruminococcus_torques_group.* Bacteroides is one of the most dominant Gram-negative bacteria in human and mammalian intestines. Lipopolysaccharide (LPS), one of the main components of its cell wall, is considered to be one of the important reasons for inducing persistent “low-grade” systemic inflammation (Li, 2009; Sang et al., 2018). As shown in Figure 6, LPS exfoliated by Bacteroidetes after death and lysis binds to LBP (LPS-binding protein, a marker of endotoxemia) and CD14 molecules on the cell surface, and further activated NF-κB signaling pathway through the TLR4 receptor on the cell membrane, promoting the expression of various pro-inflammatory factors such as TNF and IL-1β leading to systemic inflammation (Anas et al., 2010). In this study, network pharmacological studies found that Toll-like receptor signaling pathway and NF-κB signaling pathway are important pathways of FLZP in the treatment of IBS-D. TNF, IL-1β, IL-6, and INF-γ are crucial targets. In this study, the inhibitory effect of FLZP on TNF, IL-1β, IL-6, and INF-γ in IBS-D was preliminarily verified by the ELISA. After administration of FLZP, the relative abundance of Bacteroidetes increased significantly (*p* < 0.05), the levels of inflammatory cytokines TNF, IL-1β, IL-6, and INF-γ were significantly decreased (*p* < 0.05). These results suggest that FLZP can inhibit the inflammatory response in the IBS-D model by regulating the relative abundance of Bacteroidetes.

In addition, *Blautia*, *Turicibacter*, and *Ruminococcus _ _ torques _ group* may also be important gut microbiota that induce systemic inflammation. *Blautia* was found to be positively correlated with the level of IL-6 (*p* < 0.05) (Qi, 2016). In this study, Spearman correlation analysis also confirmed this correlation (*p* < 0.01). The relative abundance of *Turicibacter* was positively correlated with the level of invariant natural killer (iNK) T cells. Activated iNKT cells could produce large amounts of IL-6, IFN-γ, and other inflammatory factors (Matsuda et al., 2008; Presley et al., 2010). In this study, the relative abundance of *Turicibacter* increased in the model group (*p* < 0.01). IL-6 and IFN-γ were also significantly elevated (*p* < 0.01). *Ruminococcus__torques_group* can degrade the mucin from mucous layer on the surface of intestinal epithelial cells for proliferation, which may be an important reason that intestinal barrier is broken, accompanied with intestinal inflammation (Hoskins et al., 1985; Johansson et al., 2013; Zhu et al., 2017). After FLZP administration, the relative abundance of *Blautia*, *Turicibacter*, and *Ruminococcus_torques_group* were significantly reduced (*p* < 0.05), as were the levels of IL-6 and IFN-γ (*p* < 0.05). It suggests that FLZP can inhibit inflammation in the IBS-D model by regulating the relative abundance of *Blautia*, *Turicibacter*, and *Ruminococcus_torques_group*.

### Renovation of Intestinal Immune Barrier

*Lactobacillus* is an important member of probiotics. It is also one of the crucial gut microbiota to renovate the intestinal immune barrier. On the one hand, the proliferation of *lactobacillus* can form a biological barrier in the intestinal tract to antagonize the colonization of pathogenic microorganisms. On the other hand, *Lactobacillus* can normalize the tight junction structure between epithelial cells of the intestinal mucosa and inhibit apoptosis of epithelial cells, ultimately assisting in restoring the function of the intestinal barrier (Chen, 2017; Tang et al., 2017). Most importantly, *Lactobacillus* can regulate the immune system by signaling to communicate with the body and increase the body’s ability to fight off the adverse effects of disease.

As shown in Figure 6, *Lactobacillus reuteri ATCC PTA 6475* can suppress pro-inflammatory TNF production for anti-inflammation (Thomas et al., 2012). In this study, the network pharmacological study found that the TNF signaling pathway was an important pathway for FLZP treatment of IBS-D, and the reduction of TNF level was verified (*p* < 0.05). *Lactobacillus rhamnosus GG* (LGG) can stimulate intestinal epithelial cells to secrete APRIL by activating EGFR, thus promoting the production of intestinal anti-infection antibody lgA (Wang, 2016). IgA secretion into the gut could prevent the outgrowth of potentially harmful organisms and the maintenance of advantageous species, which was regarded as antibody-mediated immunoselection (Kubinak and Round, 2016). *Lachnospiraceae_NK4A136_group* is short chain fatty acid (SCFA)-producing bacteria (Hu et al., 2019). SCFA acetate promoted intestinal IgA responses (Wu et al., 2017). IgA responses in the gut reduced the expression of inflammation-promoting surface proteins in the *Bacteroides thetaiotamicron* (Peterson et al., 2007). Meanwhile, SCFAs inhibited inflammation through blocking the complex formation of TLR4 and LPS and also through activating AMPK/PGC1α signaling (Hu et al., 2019).

In this study, the network pharmacological study also found that the signaling pathway of the intestinal immune network for IgA production is an important pathway for FLZP in treating IBS-D. After FLZP administration, the relative abundance of *Lactobacillus* and *Lachnospiraceae_NK4A136_group* increased compared with the IBS-D model (*p* < 0.05). It suggests that FLZP can promote the renovation of intestinal immune barrier by assisting the proliferation of *Lactobacillus* and *Lachnospiraceae_NK4A136_group*.

In preliminary experiments of our study, three doses, 1.5, 1.0, and 0.5, were set to study the effect of FLZP in treating IBS-D, and results showed that there were no significant differences among the administration groups, while all three administration groups had significant differences compared to the model group. Although the efficacy intensity increased with increasing dose, there was no statistically significant difference between the three administration groups. The study was considered to explore the regulation of gut microbiota by FLZP, rather than efficacy. Therefore, in the formal experiment, the low dose group (group C) and the high dose group (group D) were selected. In addition, PCR or Western blot should be used to further verify the results of network pharmacology experiment in the follow-up study.

## Conclusion

**FIGURE 7:**
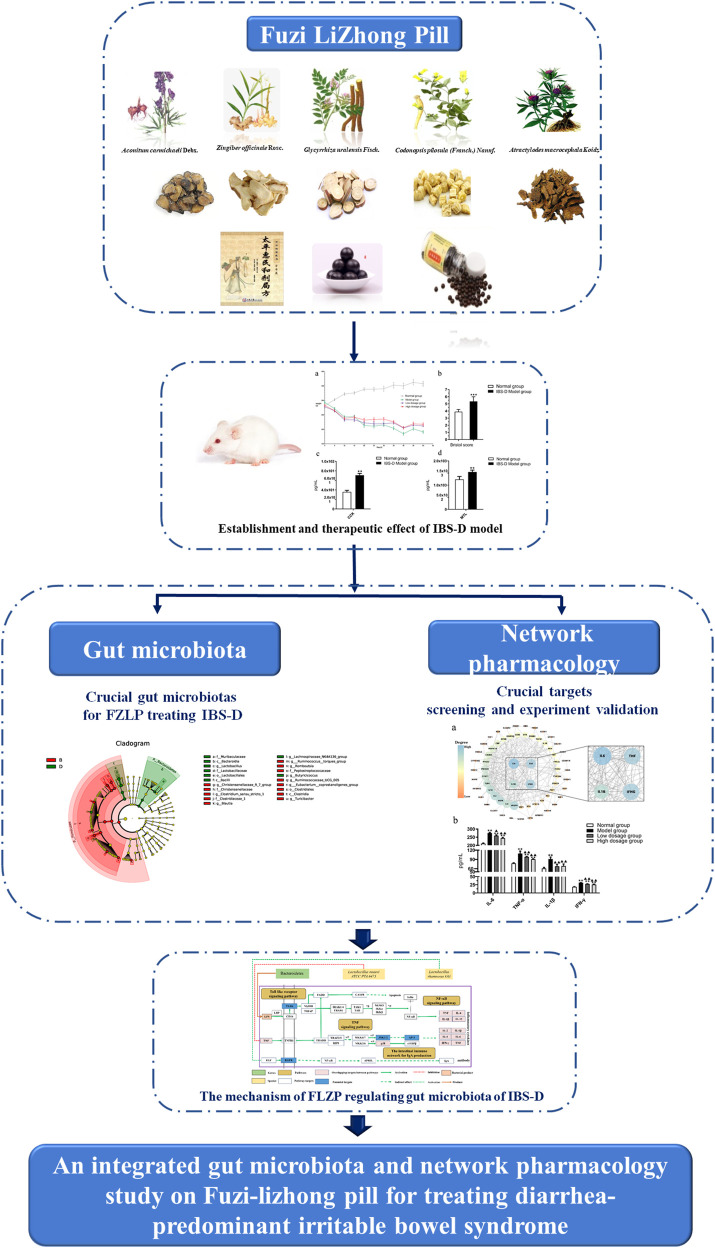
The graphical abstract of the research.

In general, eighteen gut microbiota were identified as important microbiota for FLZP in treating IBS-D. IL-6, TNF, IL-1β, and IFN-γ were crucial targets screened by network pharmacology and preliminarily verified by the ELISA. Bacteroidetes, *Blautia*, *Turicibacter*, and *Ruminococcus_torques_group* were the crucial gut microbiota that FLZP inhibits persistent systemic inflammation in the IBS-D model. *Lactobacillus* is the crucial gut microbiota that FLZP renovates intestinal immune barrier in the IBS-D model. FLZP can affect bacterial diversity and community structures in the host and regulate inflammation and the immune system to treat IBS-D. To the best of our knowledge, it was the first time to reveal the mechanism of FLZP regulating gut microbiota of IBS-D from the perspective of network pharmacology and gut microbiota (Figure 7).

## Data Availability

The data presented in the study are deposited in the NCBI BioProject repository, accession number SRP343683.
