# Supplementary material for: An Integrated Gut Microbiota and Network Pharmacology Study on Fuzi-Lizhong Pill for Treating Diarrhea-Predominant Irritable Bowel Syndrome
Source: Front Pharmacol. 2021 Nov 30;12:746923. doi: 10.3389/fphar.2021.746923 (PMC8670173; doi:10.3389/fphar.2021.746923)
Supplement: Supplementary file 1 [file DataSheet1.docx]

# Supplementary Materials

1. **Chemical Characterization Analysis of FLZP**

**1.1 Chemicals and materials**

Nine reference compounds were obtained from Sichuan Victor Biological Technology Co. Ltd.(Chengdu China). HPLC grade Ethanol, formic acid and methanol were obtained from Fisher (ThermoFisher Scientific Inc, Waltham, MA, USA). Deionised water (18 MΩ) was prepared by distilled water through a Milli-Q system (Millipore, Milford，MA, USA). Fuzi (NO. 1703003), Dangshen(NO. 1705003), Baizhu (NO. 1704088), Ganjiang (NO. 1703060) and Gancao (NO. 1703034) were purchased from Sichuan Neautus Traditional Chinese Medicine Co., Ltd. (Chengdu China) and were authenticated by Prof. Jin Pei, Department of Pharmacognosy of Chengdu University of Chinese Medicine.

**1.2 HPLC-QTOF-MS analysis condition**

Chromatographic analysis was performed in an Agilent 1290 HPLC system controlled with MassHunter Workstation Software (V B.05.00, Agilent Technologies Inc, Santa Clara, CA, USA). Samples were separated on an Agilent HC-C18 column (4.6×250 mm, 5.0 μm, Agilent Technologies Inc.) held at 35℃ and the flow rate was 1.0 mL/min with the injection volume of 10 μL. The mobile phase consisted of 0.1% formic acid-water (v/v, A) and methanol (B). The optimal gradient elution programme was as follows: 0-15 min, 95-70% A; 15-30 min,70-48% A; 30-45 min, 48-25% A; 45-48 min, 25-15% A; 48-55 min,15-2% A; and 55-65 min, 2-2% A.

Mass spectrometry was performed using an Agilent 6540 QTOF-MS (Agilent Corp., USA) equipped with a Dual AJS electrospray ionization (ESI) source, and the following operating parameters were used: positive mode, drying gas (nitrogen, N2); flow rate, 8.0 L/min; gas temperature, 325°C; nebulizer, 40 psig; sheath gas temperature, 350°C; sheath gas flow, 11 L/min; capillary voltage, 4000 V; skimmer, 65 V; OCT 1 RF Vpp, 750 V; fragmentor, 110 V. The sample collision energy was set at 10, 20, 30 and 40 V. All the operations, acquisition, and analyses of data were controlled by Agilent LCMS-QTOF Mass Hunter Acquisition Software Ver. B.06.00 (Agilent Technologies Inc.) and operated under Mass Hunter Workstation Software VersionB.06.00 (Agilent Technologies Inc.).

**1.3 Preparation of FLZP extract samples**

FLZP (1.5 g) was weighed and reflux-extracted with 50 mL 70% ethanol for 1 h. Then, the filtered supernatant sample was rotary evaporated at 40℃ to a concentration of 15 mL, and was centrifugedat 5,000 revolutions/min (rpm) for 5 min. The solution was filtered through a 0.22-μm membrane for further analysis. Identification information of constituents of FLZP was shown in Supplementary Table 1.

Table S1 Identification information of constituents of FLZP by HPLC-ESI/QTOF/MS

| Peak  No. | Rt  (min) | Systematic name | Molecular  formula | Molecular weight  (Da) | [M+H] ^+^ | | [M+Na] ^+^ | | Fragmentions(m/z) | Source |
| --- | --- | --- | --- | --- | --- | --- | --- | --- | --- | --- |
|  |  |  |  |  | **Measured mass (m/z)** | **Error(ppm)** | **Measured mass (m/z)** | **Error(ppm)** |  |  |
| 1 | 5.091 | L-Pyroglutamic acid | C_5_H_7_NO_3_ | 129.0426 | 130.0505 | 4.6136 |  |  | 130.0505[M+H]^+^,112.0123[M+H-H_2_O]^+^, 84.0449[M+H-HCOOH]^+^ | Dangshen |
| 2 | 8.051 | Codonopsine | C_14_H_21_NO_4_ | 267.1471 | 268.1543 | 0 |  |  | 268.1543[M+H]^+^, 250.1451[M+H-H_2_O]^+^ ,  205.0863[M+H-2CH_3_OH]^+^ | Dangshen |
| 3 | 9.229 | 5-hydroxymethyfurfural | C_6_H_6_O_3_ | 126.0317 | 127.0394 | 3.1486 |  |  | 127.0394[M+H]^+^, 109.0291[M+H-H_2_O]^+^ | Dangshen |
| 4 | 9.398 | Karakolidine | C_22_H_35_NO_5_ | 393.2515 | 394.2590 | 0.5072 |  |  | 394.2590[M+H]^+^, 376.2489[M+H-H_2_O]^+^, 358.2371[M+H-2H_2_O]^+^ | Fuzi |
| 5 | 10.142 | Phenylalanine | C_9_H_11_NO_2_ | 165.0790 | 166.0872 | 5.4188 |  |  | 166.0872[M+H]^+^,120.0817[M+H-HCOOH]^+^ | Dangshen |
| 6 | 11.288 | Senbusine A | C_23_H_37_NO_6_ | 423.2621 | 424.2696 | 0.4713 |  |  | 424.2696[M+H]^+^, 406.2579 [M+H-H_2_O]^+^ | Fuzi |
| 7 | 11.407 | 9-OH-senbusine A | C_23_H_37_NO_7_ | 439.2570 | 440.2635 | -1.8170 |  |  | 440.2635[M+H]^+^, 422.2531[M+H-H_2_O]^+^, 408.2318[M+H-CH_3_OH]^+^ | Fuzi |
| 8 | 12.042 | 16-β-hydroxycardiopetaline | C_21_H_33_NO_4_ | 363.2410 | 364.2480 | -0.5490 |  |  | 364.2480[M+H]^+^,346.2372[M+H-H_2_O]^+^,328.2273[M+H-2H_2_O]^+^ | Fuzi |
| 9 | 12.389 | Mesaconine | C_24_H_39_NO_9_ | 485.2625 | 486.2697 | -0.2056 |  |  | 486.2697M+H]^+^, 468.2573[M+H-H_2_O]^+^, 436.2323[M+H-H_2_O-CH_3_OH] | Fuzi |
| 10 | 12.578 | Songorine | C_22_H_31_NO_3_ | 357.2304 | 358.2382 | 1.3957 |  |  | 358.2382[M+H]^+^, 340.2267[M+H-H_2_O]^+^ | Fuzi |
| 11 | 12.908 | Karakoline | C_22_H_35_NO_4_ | 377.2566 | 378.2639 | 0 |  |  | 378.2639[M+H]^+^, 360.2533[M+H–H_2_O]^+^ | Fuzi |
| 12 | 13.081 | Isotalatizidine | C_23_H_37_NO_5_ | 407.2672 | 408.2743 | -0.2449 |  |  | 408.2743[M+H]^+^, 390.2630[M+H–H_2_O]^+^, 372.2517[M+H-2H_2_O]^+^, 358.2374[M+H–H_2_O-CH_3_OH]^+^ | Fuzi |
| 13 | 13.331 | Senbusine B | C_23_H_37_NO_6_ | 423.2621 | 424.2707 | 3.0640 |  |  | 424.2696[M+H]^+^, 406.2568[M+H-H_2_O]^+^,360.2221 [M+H-2CH_3_OH]^+^ | Fuzi |
| 14 | 13.937 | 14-acetylkarakoline | C_24_H_37_NO_5_ | 419.2672 | 420.2750 | 1.4276 |  |  | 420.2750[M+H]^+^, 402.1695[M+H-H_2_O]^+^,  356.1122[M+H-H_2_O-2CH_3_OH]^+^, | Fuzi |
| 15 | 14.091 | Aconine | C_25_H_41_NO_9_ | 499.2781 | 500.2850 | -0.7995 |  |  | 500.2850[M+H]^+^,482.2741[M+H-H_2_O]^+^, 468.2564[M+H-CH_3_OH]^+^, 450.2478[M+H–H_2_O-CH_3_OH]^+^, 436.2309[M+H-2CH_3_OH]^+^, 418.2209[M+H–H_2_O-2CH_3_OH]^+^ | Fuzi |
| 16 | 14.380 | Hetisine | C_20_H_27_NO_3_ | 329.1991 | 330.2064 | 0 |  |  | 330.2064[M+H]^+^, 312.1951[M+H-H_2_O]^+^ | Fuzi |
| 17 | 15.319 | Hypaconine | C_24_H_39_NO_8_ | 469.2676 | 470.2744 | -0.8506 |  |  | 470.2744[M+H]^+^, 453.2301[M+H-OH]^+^, 438.2474[M+H-CH_3_OH]^+^, 406.2212[M+H-2CH_3_OH]^+^, 374.1941[M+H-3CH_3_OH]^+^ | Fuzi |
| 18 | 15.810 | Fuzitine | C_20_H_23_NO_4_ | 341.1627 | 342.1697 | -0.8767 |  |  | 342.1697[M+H]^+^, 324.1026[M+H-H_2_O]^+^ | Fuzi |
| 19 | 16.070 | Fuziline | C_24_H_39_NO_7_ | 453.2727 | 454.2800 | 0.2201 |  |  | 454.2800[M+H]^+^, 436.2677[M+H-H_2_O]^+^, 418.2583[M+H-2H_2_O]^+^, 404.2443[M+H-H_2_O-CH_3_OH]^+^, 386.2295[M+H-2H_2_O-CH_3_OH]^+^, 354.2069[M+H-2H_2_O-2CH_3_OH]^+^ | Fuzi |
| 20 | 16.248 | Tau-cadinol | C_15_H_26_O | 222.1984 | 245.1852 | -9.7884 |  |  | 245.1852[M+H]^+^, 213.0195[M+H-CH_3_OH]^+^, 199.1252[M+H-CH_3_OH-CH_3_]^+^, 184.9885[M+H-CH_3_OH-2CH_3_] ^+^，  169.0055[M+H-CH_3_OH-3CH_3_]^+^， | Ganjiang |
| 21 | 16.573 | Neoline | C_24_H_39_NO_6_ | 437.2777 | 438.2848 | -0.4563 |  |  | 438.2848M+H]^+^, 420.2756[M+H-H_2_O]^+^, 388.2478[M+H-H_2_O-CH_3_OH]^+^, 370.2365[M+H-2H_2_O-CH_3_OH]^+^, 356.2213[M+H-H_2_O-2CH_3_OH]^+^ | Fuzi |
| 22 | 16.743 | Talatisamine | C_24_H_39_NO_5_ | 421.2828 | 422.2899 | 0.4736 |  |  | 422.2899[M+H]^+^, 390.2621[M+H-CH_3_OH]^+^, 358.2349[M+H-2CH_3_OH]^+^ | Fuzi |
| 23 | 18.651 | Chasmanine | C_25_H_41_NO_6_ | 451.2934 | 452.3008 | 02210 |  |  | 452.3008[M+H]^+^, 420.2737[M+H-CH_3_OH]^+^ | Fuzi |
| 24 | 19.739 | Geranial | C_10_H_16_O | 152.1201 | 153.1275 | 0.6530 |  |  | 153.1275[M+H]^+^, 135.1162[M+H-H_2_O]^+^, 125.0940[M+H-CO]^+^ | Ganjiang |
| 25 | 20.390 | 14-Acetyltalatizamine | C_26_H_41_NO_6_ | 463.2934 | 464.3014 | 1.5076 |  |  | 464.3014[M+H]^+^, 432.2753[M+H-CH_3_OH]^+^, 414.2645[M+H-CH_3_OH-H_2_O]^+^, 400.2486[M+H-2CH_3_OH]^+^, 372.2522[M+H-CH_3_OH-AcOH]^+^ | Fuzi |
| 26 | 21.828 | 7-hydroxycoumarin | C_9_H_6_O_3_ | 162.0317 | 163.0395 | 3.0667 |  |  | 163.0395[M+H]^+^, 145.0627[M+H-H_2_O]^+^，135.0453[M+H-CO]^+^ | Baizhu |
| 27 | 23.891 | Schaftoside | C_26_H_28_O_14_ | 564.1479 | 565.1542 | -1.7694 |  |  | 565.1542[M+H]^+^, 547.1434[M+H-H_2_O]^+^, 529.1303[M+H-2H_2_O]^+^, 511.1220[M+H-3H_2_O]^+^ | Gancao |
| 28 | 24.041 | Scopoletin | C_10_H_8_O_4_ | 192.0423 | 193.0500 | 2.5900 |  |  | 193.0500[M+H]^+^, 161.0603[M+H-CH_3_OH]^+^ | Baizhu |
| 29# | 24.785 | Liquiritigenin | C_15_H_12_O_4_ | 256.0736 | 257.0819 | 4.2788 |  |  | 257.0819[M+H]^+^, 239.0707[M+H-H_2_O]^+^, 137.0235[C_7_H_4_O_3_+H]^+^, 121.0280[C_8_H_8_O+H]^+^ ,119.0491[C_8_H_8_O-H]^+^ | Gancao |
| 30# | 27.065 | Benzoylmesaconine | C_31_H_43_NO_10_ | 589.2887 | 590.2959 | -0.1694 |  |  | 590.2959[M+H]^+^,572.2826[M+H-H_2_O]^+^, 558.2663[M+H-CH_3_OH]^+^ 540.2573[M+H-CH_3_OH-H_2_O]^+^ | Fuzi |
| 31 | 27.325 | Isoviolanthin | C_27_H_30_O_14_ | 578.1636 | 579.1700 | -1.3812 |  |  | 579.1700[M+H]^+^,561.1588[M+H-H_2_O]^+^, 543.1485[M+H-2H_2_O]^+^, 525.1382[M+H-3H_2_O]^+^ | Gancao |
| 32# | 27.614 | Benzoylaconine | C_32_H_45_NO_10_ | 603.3043 | 604.3114 | -0.3309 |  |  | 604.3114[M+H]^+^,587.2801[M+H-OH]^+^, 554.2711[M+H-2CH_3_OH]^+^ | Fuzi |
| 33# | 28.595 | Benzoylhypaconine | C_31_H_43_NO_9_ | 573.2938 | 574.3011 | 0 |  |  | 574.3011[M+H]^+^, 542.2745[M+H-CH_3_OH]^+^,，510.2457[M+H-2CH_3_OH]^+^ | Fuzi |
| 34 | 28.748 | Lobetyolinin | C_26_H_38_O_13_ | 558.2312 |  |  | 581.2203 | -0.3441 | 581.2203[M+Na]^+^, 419.1709[M+Na-C_6_H_10_O_5_]^+^ | Dangshen |
| 35 | 31.019 | Liquiritin apioside or Isoliquiritin apioside | C_26_H_30_O_13_ | 550.1686 | 551.1751 | -1.4514 |  |  | 551.1751[M+H]^+^, 419.1333[M+H-(Apiose-H_2_O)]^+^, 257.0830[M+H-(Apiose-H_2_O)-(Glc-H_2_O)]^+^ | Gancao |
| 36# | 31.163 | Mesaconitine | C_33_H_45_NO_11_ | 631.2993 | 632.3067 | 0.3163 |  |  | 632.3067[M+H]^+^, 614.1110[M+H-H_2_O]^+^,  600.2748[M+H-CH_3_OH]^+^, 572.2834[M+H-AcOH]^+^ | Fuzi |
| 37 | 31.423 | 7-methoxy-liquiritin | C_22_H_22_O_9_ | 430.1264 | 431.1332 | -1.1597 |  |  | 431.1332[M+H]^+^,269.0811[M+H-(Glc-H_2_O)]^+^ | Gancao |
| 38 | 31.646 | 14-Benzoylneoline | C_31_H_43_NO_7_ | 541.3040 | 542.3135 | 4.2411 |  |  | 542.3135[M+H]^+^,524.3010[M+H-H_2_O]^+^,  510.2731[M+H-CH_3_OH]^+^, 492.2733[M+H-H_2_O-CH_3_OH]^+^ | Fuzi |
| 39 | 31.659 | Dehydrated benzoylhypaconine | C_31_H_41_NO_8_ | 555.2832 | 556.2906 | 0.1798 |  |  | 556.2906[M+H]^+^,524.2647[M+H-CH_3_OH]^+^, 492.2381[M+H-2CH_3_OH]^+^ | Fuzi |
| 40 | 31.683 | Liquiritin or Isoliquiritin | C_21_H_22_O_9_ | 418.1264 | 419.1335 | 0.4771 |  |  | 419.1335[M+H]^+^, 257.0811[M+H-(Glc-H_2_O)]^+^ | Gancao |
| 41 | 31.921 | Aconifine | C_34_H_47_NO_12_ | 661.3098 | 662.3172 | 0.1509 |  |  | 662.3172[M+H]^+^, 644.3095[M+H-H_2_O]^+^,  626.1346 [M+H-2H_2_O]^+^ | Fuzi |
| 42 | 32.100 | Hypaconitine | C_33_H_45_NO_10_ | 615.3043 | 616.3116 | 0 |  |  | 616.3116[M+H]^+^, 584.2843[M+H-CH_3_OH]^+^  556.2899[M+H-C_2_H_4_O_2_]^+^ , 524.2533[M+H-C_2_H_4_O_2_-CH_3_OH]^+^ ,496.2678[M+H-C_2_H_4_O_2_-CH_3_OH-CO]^+^ | Fuzi |
| 43 | 32.245 | Formononetin | C_16_H_12_O_4_ | 268.0736 | 269.0814 | 2.2298 |  |  | 269.0814[M+H]^+^, 254.0580[M+H-CH_3_]^+^, 237.0536[M+H-CH_3_OH]^+^,225.0554[M+H-CH_3_-CO]^+^, 213.0908[M+H-C_2_O_2_]^+^, 181.0666[M+H-C_2_O_2_-CH_3_OH]^+^ | Gancao |
| 44 | 32.528 | Aconitine | C_34_H_47_NO_11_ | 645.3149 | 646.3216 | -0.9283 |  |  | 646.3216[M+H]^+^, 628.3140[M+H-H_2_O]^+^,  596.2849[M+H-H_2_O-CH_3_OH]^+^ | Fuzi |
| 45 | 33.241 | Deoxyaconitine | C_34_H_47_NO_10_ | 629.3200 | 630.3273 | 0 |  |  | 630.3273[M+H]^+^, 598.3070[M+H-CH_3_OH]^+^ | Fuzi |
| 46 | 36.853 | Echinatin | C_16_H_14_O_4_ | 270.0892 | 271.0963 | -0.7377 |  |  | 271.0963[M+H]^+^,253.0850[M+H-H_2_O]^+^ | Gancao |
| 47 | 38.085 | Benzoic acid | C_7_H_6_O_2_ | 122.0368 | 123.0447 | 4.8763 |  |  | 123.0447[M+H]^+^, 77.0379[M+H-HCOOH]^+^ | Baizhu |
| 48# | 39.763 | Isoliquiritigenin | C_15_H_12_O_4_ | 256.0736 | 257.0814 | 2.334 |  |  | 257.0814[M+H]^+^, 239.0704[M+H-H_2_O]^+^, 137.0235[C_7_H_4_O_3_+H]^+^,121.0277[C_8_H_8_O+H]^+^,120.0527 [C_7_H_4_O_3_+H-OH]^+^ | Gancao |
| 49 | 40.720 | Glycycoumarin | C_21_H_20_O_6_ | 368.1260 | 369.1345 | 3.2508 |  |  | 369.1345[M+H]^+^, 333.2235[M+H-2H_2_O]^+^,  313.1057 [M+H-C_4_H_8_]^+^, | Gancao |
| 50 | 41.513 | 6-gingerdione | C_17_H_24_O_4_ | 292.1675 | 293.1736 | -2.7520 |  |  | 293.1736[M+H]^+^, 275.1650[M+H-H_2_O]^+^  257.1517[M+H-2H_2_O]^+^ | Ganjiang |
| 51 | 42.593 | Kumatakenin | C_17_H_14_O_6_ | 314.0790 | 315.0859 | -1.2694 |  |  | 315.0859[M+H]^+^,298.2146[M+H-OH]^+^,  279.0782[M+H-2H_2_O]^+^ | Ganjiang |
| 52 | 43.486 | 6-gingerol | C_17_H_26_O_4_ | 294.1831 |  |  | 317.1737 | 4.4140 | 317.1771[M+Na]^+^, 299.2546[M+Na-H_2_O]^+^ | Ganjiang |
| 53 | 43.507 | Gingerenone-A | C_21_H_24_O_5_ | 356.1624 | 357.1710 | 3.6397 |  |  | 357.1710[M+H]^+^, 339.2718[M+H-H_2_O]^+^, 321.2612[M+H-2H_2_O]^+^ | Ganjiang |
| 54 | 43.544 | 6-shogaol | C_17_H_24_O_3_ | 276.1725 | 277.1795 | 1.0823 |  |  | 277.1795[M+H]^+^, 259.1694[M+Na-H_2_O]^+^, | Ganjiang |
| 55 | 45.779 | Lupiwighteone | C_20_H_18_O_5_ | 338.1154 | 339.1239 | 3.5385 |  |  | 339.1239[M+H]^+^, 321.2818[M+H-H_2_O]^+^ | Gancao |
| 56 | 46.339 | Atractylenolide Ⅲ | C_15_H_20_O_3_ | 248.1412 | 245.1485 | 0 |  |  | 249.1485[M+H]^+^, 231.1389[M+H-H_2_O]^+^, 175.0751[M+H-H_2_O-2CO]^+^, 163.0756[M+H-H_2_O-C_5_H_8_]^+^ | Baizhu |
| 57 | 48.364 | Gancaonin L | C_20_H_18_O_6_ | 354.1103 | 355.1189 | 3.6607 |  |  | 355.1189[M+H]^+^, 337.2536[M+H-H_2_O]^+^ | Gancao |
| 58 | 48.398 | Licoricesaponin G2 | C_42_H_62_O_17_ | 838.3987 | 839.4076 | 1.9061 |  |  | 839.4076[M+H]^+^, 663.3722[M+H-(GluA-H_2_O)]^＋^, 469.3308[M+H-2(GluA-H_2_O)-H_2_O]^+^ | Gancao |
| 59# | 48.887 | Atractylenolide Ⅱ | C_15_H_20_O_2_ | 232.1463 | 233.1541 | 2.1445 |  |  | 233.1541[M+Na]^+^, 187.1485[M+Na-CH_2_O_2_]^+^,  159.0806[M+Na-CH_2_O_2_-C_2_H_4_]^+^, 145.1013 [M+Na-CH_2_O_2_-C_3_H_6_]^+^ ,131.0857[M+Na-CH_2_O_2_-C_4_H_8_]^+^ , 105.0703[M+Na-CH_2_O_2_-C_4_H_8_-C_2_H_2_]^+^ | Baizhu |
| 60# | 49.296 | Glycyrrhizic acid | C_42_H_62_O_16_ | 822.4038 | 823.4130 | 2.3075 |  |  | 823.4130 [M+H]^+^, 647.3793[M+H-(GluA-H_2_O)]^+^ | Gancao |
| 61 | 49.667 | Farnesal | C_15_H_24_O | 220.1827 | 221.1907 | 3.1647 |  |  | 221.1907M+H]^+^,192.9740[M+H-CO]^+^ | Ganjiang |
| 62# | 49.841 | Glycyrrhetinic acid | C_30_H_46_O_4_ | 470.3396 | 471.3488 | 4.031 |  |  | 471.3488[M+H]^+^, 453.3354[M+H-H_2_O]^+^, 435.3224[M+H-2H_2_O]^+^,425.3378[M+H-HCOOH]^+^ | Gancao |
| 63 | 50.671 | Licorice saponin B2 | C_42_H_64_O_15_ | 808.4245 |  |  | 831.4151 | 1.6838 | 831.4151 [M+Na]^+^,655.3825[M+Na-(GluA-H_2_O)]^+^,  479.3547[M+Na-2(GluA-H_2_O)]^+^ | Gancao |
| 64 | 51.232 | Licoricone | C_22_H_22_O_6_ | 382.1416 | 383.1502 | 3.3929 |  |  | 383.1502[M+H]^+^, 355.1587[M+H-C_2_H_4_]^+^ | Gancao |
| 65 | 51.390 | Atractylenolide Ⅰ | C_15_H_18_O_2_ | 230.1307 | 231.1383 | 1.2979 |  |  | 231.1383[M+H]^+^, 185.1326[M+H-HCOOH]^+^ ，  157.1012[M+H-HCOOH-C_2_H_4_]^+^,105.0701 [M+H-HCOOH-2C_2_H_4_-2C]^+^ | Baizhu |
| 66 | 52.950 | Neoglycyrol | C_21_H_18_O_6_ | 366.1103 | 367.1165 | -0.5447 |  |  | 367.1165[M+H]^+^, 349.2239[M+H-H_2_O]^+^，  335.2389[M+H-CH_3_OH]^+^，  317.2283[M+H-2H_2_O- CH_3_OH]^+^ | Gancao |
| 67 | 54.310 | Licorice-saponin J2 | C_42_H_64_O_16_ | 824.4194 | 825.4286 | 2.3018 |  |  | 825.4286[M+H]^+^, 649.3906 [M+H-(GluA-H_2_O)]^+^, 455.3537[M+H-2(GluA-H_2_O)-H_2_O]^+^ ,437.3435 [M+H-2(GluA-H_2_O)-2H_2_O]^+^ | Gancao |

^#^ Indicates compounds identified by comparing with the reference standards.

**2. quality control of FZLP**

**2.1 HPLC conditions of quantification of liquiritin and glycyrrhizin**

The Thermo Scientific Ultimate 300 High-Performance Liquid Chromatography instrument (ThermoFisher Scientific, USA) was used to analyze the extract solution and chromatographic separation was per-formed using an Thermo Scientific™ Accucore™ C18 Column (100 ×3 mm, 2.6 μm; ThermoFisher Scientific, USA) with a column temperature of 25 ℃. A gradient- elution was provided with 0.05% phosphoric acid water as solvent A and acetonitrile as solvent B, using a flow rate of 1.0 mL/min, where 10 μL samples were injected and the effluent absorbance was measured at 237 nm. The chromatographic separation was conducted by the following gradient program: 0-8 min (19% B), 8–35 min (19–50% B), 35–36min (50–100% B), 36–40min (100–19% B), 40–45min (19% B).

**2.2 UPLC-QQQ-MS conditions of quantification of benzoylmesaconine, benzoylaconine and benzoylhypaconine**

Chromatographic separation was achieved on a Agilent HC-C18 Column (2.1mm×100mm，1.8 um). The mobile phase contained A (0.05% formic acid) and B (acetonitrile) with a flow rate of 0.2 mL/min. The chromatographic separation was conducted by the following gradient program: 0-8 min (15-30% B), 8–15 min (30–54.5% B). The column temperature was maintained at 25 ℃. The injection volume was 5 µL and a 5 min equilibrating was setting before each injection.

A TSQ-Fortis Field triple quadrupole mass spectrometer (ThermoFisher Scientific, USA) was used to perform positive ion monitoring and mass spectrometry in selective reaction monitoring (SRM) mode. The parameters of ion source were as follows: capillary voltage +4000V, dryer temperature 350℃, ion transfer tube temperature 300℃, sheath flow rate 35Arb, auxiliary gas flow rate 10Arb, tail blowing flow rate 1Arb. Supplementary Table 1 provided SRM parameter information for the three substances to be measured.

Table S2 SRM parameter information of three compounds.

| compound | quantitative ion pairs | Cracking voltage (V) | Collision energy (eV) | |
| --- | --- | --- | --- | --- |
| benzoylmesaconine | 590/540.220/105.000 | 30 | | 45 |
| benzoylaconine | 604/554.274104.99 | 30 | | 45 |
| benzoylhypaconine | 574/542.230/104.946 | 40 | | 45 |

**2.3 Preparation of calibration curves and FZLP sample**

The standard stock solutions of liquiritin (256 µg/ml), glycyrrhizin (540 µg/ml), benzoylmesaconine (17.5 µg/ml), benzoylaconine (2.3 µg/ml) and benzoylhypaconine (1.25 µg/ml) were separately prepared in methanol. A mixed Liquiritin and glycyrrhizin standard solution was used in HPLC. A mixed Benzoylmesaconine, benzoylaconine and benzoylhypaconine standard solution was used in UPLC-QQQ-MS. Then aliquots of these solutions were further diluted with methanol to a series of concentrations for the construction of calibration curves.

The fragmentized FLZP was accurately weighed and extracted with 70% ethanol (weight/volume, 1:100) by conduct ultrasonic treatment (power 300W, frequency 25kHz) for 45 minutes at ambient temperature. The final sample was then filtered through a syringe filter (0.22 µm) before injecting. The quality control results are shown in Supplementary Table 2 and Supplementary Figures 1 and 2.

Table S3 Retention time, regression equations, correlation coefficient and linear

ranges of the five compounds

| compound | retention time(min) | calibration curves | R^2^ | linear rang(µg/ml) |
| --- | --- | --- | --- | --- |
| liquiritin | 8.43 | Y=0.2704X+1.1634 | 0.9995 | 8.000-256.0 |
| glycyrrhizin | 30.32 | Y=0.0832X+0.5176 | 0.9994 | 16.88-540.0 |
| benzoylmesaconine | 7.58 | Y=2e+06X+85703 | 0.9980 | 0.5469-17.50 |
| benzoylaconine | 8.66 | Y=4e+06X+48210 | 0.9983 | 0.0719-2.300 |
| benzoylhypaconine | 9.35 | Y=606362X+472102 | 0.9933 | 0.0391-1.250 |


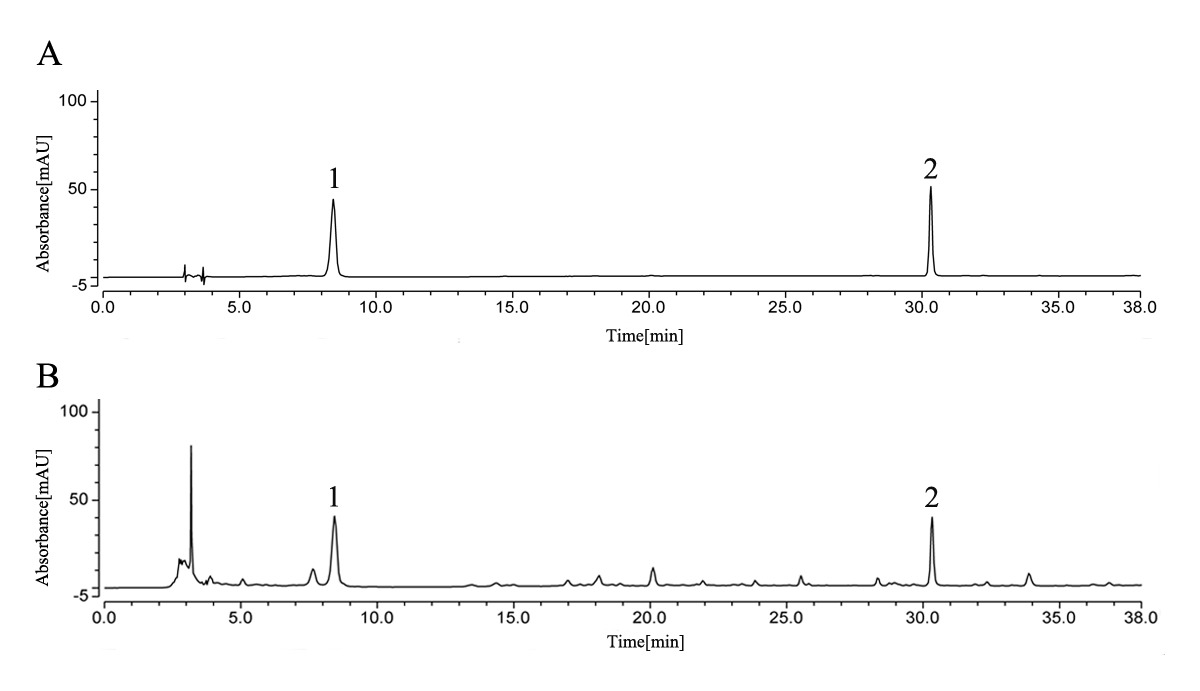


Fig. S1 HPLC chromatograms of standard solution (A) and FLZP sample (B). (1: liquiritin, 2: glycyrrhizin)


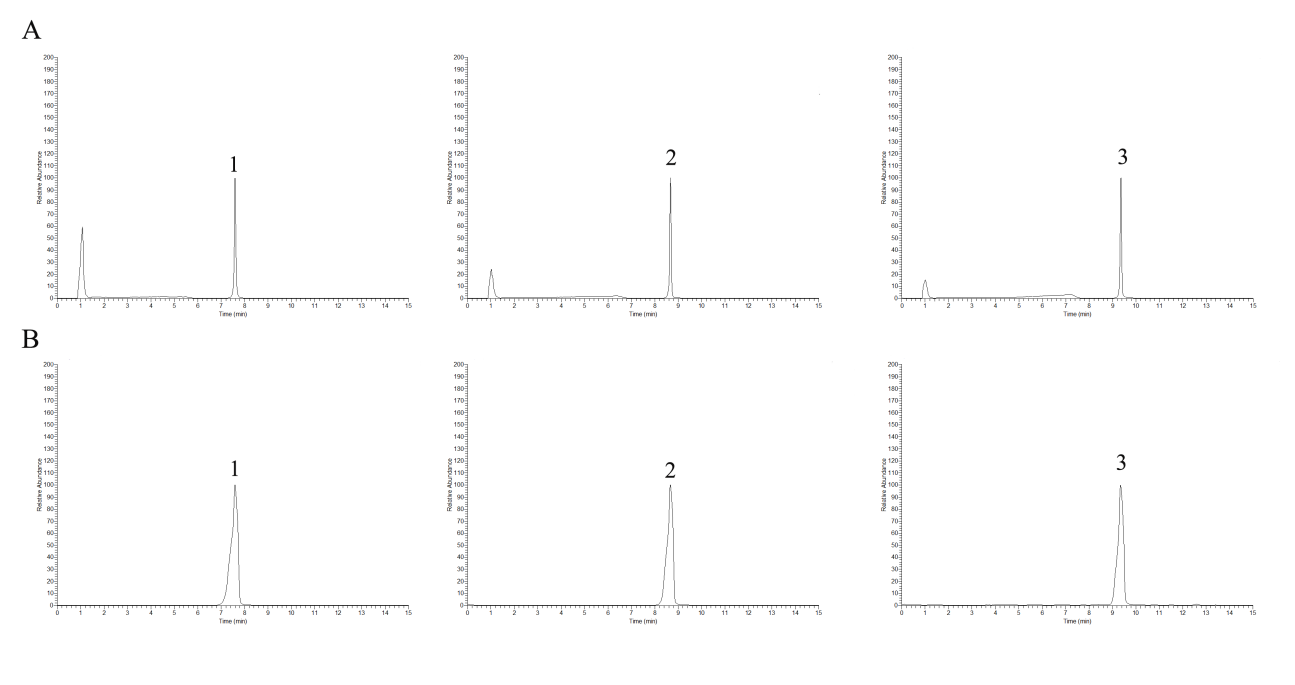


Fig. S2 UPLC-QQQ-MS chromatograms of standard solution (A) and FLZP sample (B). (1: benzoylmesaconine, 2: benzoylaconine, 3: benzoylhypaconine)

Table S4 Fecal character Bristol classification and scoring criteria

| Grade | Fecal traits | Score |
| --- | --- | --- |
| 1 | Dispersed similar to dry balls and nuts and was difficult to discharge | 1 |
| 2 | Sausage-shaped and multipiece | 2 |
| 3 | Sausage-like with a cracked surface | 3 |
| 4 | Sausage-like or serpentine, smooth and soft | 4 |
| 5 | Soft clump with clear edges and was easy to discharge | 5 |
| 6 | A soft "sheet" with hairy edges or mushy consistency | 6 |
| 7 | Watery stool with no solid compounds | 7 |

Table S5 Informations of 143 potential components of FLZP

| MOL ID | Compound | MW | OB (%) | DL | Source |
| --- | --- | --- | --- | --- | --- |
| MOL002464 | 1-Monolinolein | 354.59 | 37.18 | 0.3 | *Zingiber officinale* Rosc. |
| MOL002501 | [(1S)-3-[(E)-but-2-enyl]-2-methyl-4-oxo-1-cyclopent-2-enyl] (1R,3R)-3-[(E)-3-methoxy-2-methyl-3-oxoprop-1-enyl]-2,2-dimethylcyclopropane-1-carboxylate | 360.49 | 62.52 | 0.31 | *Zingiber officinale* Rosc. |
| MOL002514 | Sexangularetin | 316.28 | 62.86 | 0.3 | *Zingiber officinale* Rosc. |
| MOL000358 | beta-sitosterol | 414.79 | 36.91 | 0.75 | *Zingiber officinale* Rosc. |
| MOL001484 | Inermine | 284.28 | 75.18 | 0.54 | *Glycyrrhiza uralensis* Fisch. |
| MOL001792 | DFV | 256.27 | 32.76 | 0.18 | *Glycyrrhiza uralensis* Fisch. |
| MOL000211 | Mairin | 456.78 | 55.38 | 0.78 | *Glycyrrhiza uralensis* Fisch. |
| MOL002311 | Glycyrol | 366.39 | 90.78 | 0.67 | *Glycyrrhiza uralensis* Fisch. |
| MOL000239 | Jaranol | 314.31 | 50.83 | 0.29 | *Glycyrrhiza uralensis* Fisch. |
| MOL002565 | Medicarpin | 270.3 | 49.22 | 0.34 | *Glycyrrhiza uralensis* Fisch. |
| MOL000354 | isorhamnetin | 316.28 | 49.6 | 0.31 | *Glycyrrhiza uralensis* Fisch. |
| MOL003656 | Lupiwighteone | 338.38 | 51.64 | 0.37 | *Glycyrrhiza uralensis* Fisch. |
| MOL000392 | formononetin | 268.28 | 69.67 | 0.21 | *Glycyrrhiza uralensis* Fisch. |
| MOL000417 | Calycosin | 284.28 | 47.75 | 0.24 | *Glycyrrhiza uralensis* Fisch. |
| MOL000422 | kaempferol | 286.25 | 41.88 | 0.24 | *Glycyrrhiza uralensis* Fisch. |
| MOL004328 | naringenin | 272.27 | 59.29 | 0.21 | *Glycyrrhiza uralensis* Fisch. |
| MOL004805 | (2S)-2-[4-hydroxy-3-(3-methylbut-2-enyl)phenyl]-8,8-dimethyl-2,3-dihydropyrano[2,3-f]chromen-4-one | 390.51 | 31.79 | 0.72 | *Glycyrrhiza uralensis* Fisch. |
| MOL004806 | euchrenone | 406.56 | 30.29 | 0.57 | *Glycyrrhiza uralensis* Fisch. |
| MOL004808 | glyasperin B | 370.43 | 65.22 | 0.44 | *Glycyrrhiza uralensis* Fisch. |
| MOL004810 | glyasperin F | 354.38 | 75.84 | 0.54 | *Glycyrrhiza uralensis* Fisch. |
| MOL004811 | Glyasperin C | 356.45 | 45.56 | 0.4 | *Glycyrrhiza uralensis* Fisch. |
| MOL004814 | Isotrifoliol | 298.26 | 31.94 | 0.42 | *Glycyrrhiza uralensis* Fisch. |
| MOL004815 | (E)-1-(2,4-dihydroxyphenyl)-3-(2,2-dimethylchromen-6-yl)prop-2-en-1-one | 322.38 | 39.62 | 0.35 | *Glycyrrhiza uralensis* Fisch. |
| MOL004820 | kanzonols W | 336.36 | 50.48 | 0.52 | *Glycyrrhiza uralensis* Fisch. |
| MOL004824 | (2S)-6-(2,4-dihydroxyphenyl)-2-(2-hydroxypropan-2-yl)-4-methoxy-2,3-dihydrofuro[3,2-g]chromen-7-one | 384.41 | 60.25 | 0.63 | *Glycyrrhiza uralensis* Fisch. |
| MOL004827 | Semilicoisoflavone B | 352.36 | 48.78 | 0.55 | *Glycyrrhiza uralensis* Fisch. |
| MOL004828 | Glepidotin A | 338.38 | 44.72 | 0.35 | *Glycyrrhiza uralensis* Fisch. |
| MOL004829 | Glepidotin B | 340.4 | 64.46 | 0.34 | *Glycyrrhiza uralensis* Fisch. |
| MOL004833 | Phaseolinisoflavan | 324.4 | 32.01 | 0.45 | *Glycyrrhiza uralensis* Fisch. |
| MOL004835 | Glypallichalcone | 284.33 | 61.6 | 0.19 | *Glycyrrhiza uralensis* Fisch. |
| MOL004838 | 8-(6-hydroxy-2-benzofuranyl)-2,2-dimethyl-5-chromenol | 308.35 | 58.44 | 0.38 | *Glycyrrhiza uralensis* Fisch. |
| MOL004841 | Licochalcone B | 286.3 | 76.76 | 0.19 | *Glycyrrhiza uralensis* Fisch. |
| MOL004848 | licochalcone G | 354.43 | 49.25 | 0.32 | *Glycyrrhiza uralensis* Fisch. |
| MOL004849 | 3-(2,4-dihydroxyphenyl)-8-(1,1-dimethylprop-2-enyl)-7-hydroxy-5-methoxy-coumarin | 368.41 | 59.62 | 0.43 | *Glycyrrhiza uralensis* Fisch. |
| MOL004855 | Licoricone | 382.44 | 63.58 | 0.47 | *Glycyrrhiza uralensis* Fisch. |
| MOL004856 | Gancaonin A | 352.41 | 51.08 | 0.4 | *Glycyrrhiza uralensis* Fisch. |
| MOL004857 | Gancaonin B | 368.41 | 48.79 | 0.45 | *Glycyrrhiza uralensis* Fisch. |
| MOL004860 | licorice glycoside E | 693.71 | 32.89 | 0.27 | *Glycyrrhiza uralensis* Fisch. |
| MOL004863 | 3-(3,4-dihydroxyphenyl)-5,7-dihydroxy-8-(3-methylbut-2-enyl)chromone | 354.38 | 66.37 | 0.41 | *Glycyrrhiza uralensis* Fisch. |
| MOL004864 | 5,7-dihydroxy-3-(4-methoxyphenyl)-8-(3-methylbut-2-enyl)chromone | 352.41 | 30.49 | 0.41 | *Glycyrrhiza uralensis* Fisch. |
| MOL004866 | 2-(3,4-dihydroxyphenyl)-5,7-dihydroxy-6-(3-methylbut-2-enyl)chromone | 354.38 | 44.15 | 0.41 | *Glycyrrhiza uralensis* Fisch. |
| MOL004879 | Glycyrin | 382.44 | 52.61 | 0.47 | *Glycyrrhiza uralensis* Fisch. |
| MOL004882 | Licocoumarone | 340.4 | 33.21 | 0.36 | *Glycyrrhiza uralensis* Fisch. |
| MOL004883 | Licoisoflavone | 354.38 | 41.61 | 0.42 | *Glycyrrhiza uralensis* Fisch. |
| MOL004884 | Licoisoflavone B | 352.36 | 38.93 | 0.55 | *Glycyrrhiza uralensis* Fisch. |
| MOL004885 | licoisoflavanone | 354.38 | 52.47 | 0.54 | *Glycyrrhiza uralensis* Fisch. |
| MOL004891 | shinpterocarpin | 322.38 | 80.3 | 0.73 | *Glycyrrhiza uralensis* Fisch. |
| MOL004898 | (E)-3-[3,4-dihydroxy-5-(3-methylbut-2-enyl)phenyl]-1-(2,4-dihydroxyphenyl)prop-2-en-1-one | 340.4 | 46.27 | 0.31 | *Glycyrrhiza uralensis* Fisch. |
| MOL004903 | liquiritin | 418.43 | 65.69 | 0.74 | *Glycyrrhiza uralensis* Fisch. |
| MOL004904 | licopyranocoumarin | 384.41 | 80.36 | 0.65 | *Glycyrrhiza uralensis* Fisch. |
| MOL004905 | 3,22-Dihydroxy-11-oxo-delta(12)-oleanene-27-alpha-methoxycarbonyl-29-oic acid | 512.75 | 34.32 | 0.55 | *Glycyrrhiza uralensis* Fisch. |
| MOL004907 | Glyzaglabrin | 298.26 | 61.07 | 0.35 | *Glycyrrhiza uralensis* Fisch. |
| MOL004908 | Glabridin | 324.4 | 53.25 | 0.47 | *Glycyrrhiza uralensis* Fisch. |
| MOL004910 | Glabranin | 324.4 | 52.9 | 0.31 | *Glycyrrhiza uralensis* Fisch. |
| MOL004911 | Glabrene | 322.38 | 46.27 | 0.44 | *Glycyrrhiza uralensis* Fisch. |
| MOL004912 | Glabrone | 336.36 | 52.51 | 0.5 | *Glycyrrhiza uralensis* Fisch. |
| MOL004913 | 1,3-dihydroxy-9-methoxy-6-benzofurano[3,2-c]chromenone | 298.26 | 48.14 | 0.43 | *Glycyrrhiza uralensis* Fisch. |
| MOL004914 | 1,3-dihydroxy-8,9-dimethoxy-6-benzofurano[3,2-c]chromenone | 328.29 | 62.9 | 0.53 | *Glycyrrhiza uralensis* Fisch. |
| MOL004915 | Eurycarpin A | 338.38 | 43.28 | 0.37 | *Glycyrrhiza uralensis* Fisch. |
| MOL004917 | glycyroside | 562.57 | 37.25 | 0.79 | *Glycyrrhiza uralensis* Fisch. |
| MOL004924 | (-)-Medicocarpin | 432.46 | 40.99 | 0.95 | *Glycyrrhiza uralensis* Fisch. |
| MOL004935 | Sigmoidin-B | 356.4 | 34.88 | 0.41 | *Glycyrrhiza uralensis* Fisch. |
| MOL004941 | (2R)-7-hydroxy-2-(4-hydroxyphenyl)chroman-4-one | 256.27 | 71.12 | 0.18 | *Glycyrrhiza uralensis* Fisch. |
| MOL004945 | (2S)-7-hydroxy-2-(4-hydroxyphenyl)-8-(3-methylbut-2-enyl)chroman-4-one | 324.4 | 36.57 | 0.32 | *Glycyrrhiza uralensis* Fisch. |
| MOL004948 | Isoglycyrol | 366.39 | 44.7 | 0.84 | *Glycyrrhiza uralensis* Fisch. |
| MOL004949 | Isolicoflavonol | 354.38 | 45.17 | 0.42 | *Glycyrrhiza uralensis* Fisch. |
| MOL004957 | HMO | 268.28 | 38.37 | 0.21 | *Glycyrrhiza uralensis* Fisch. |
| MOL004959 | 1-Methoxyphaseollidin | 354.43 | 69.98 | 0.64 | *Glycyrrhiza uralensis* Fisch. |
| MOL004961 | Quercetin der. | 330.31 | 46.45 | 0.33 | *Glycyrrhiza uralensis* Fisch. |
| MOL004966 | 3'-Hydroxy-4'-O-Methylglabridin | 354.43 | 43.71 | 0.57 | *Glycyrrhiza uralensis* Fisch. |
| MOL000497 | licochalcone a | 338.43 | 40.79 | 0.29 | *Glycyrrhiza uralensis* Fisch. |
| MOL004974 | 3'-Methoxyglabridin | 354.43 | 46.16 | 0.57 | *Glycyrrhiza uralensis* Fisch. |
| MOL004978 | 2-[(3R)-8,8-dimethyl-3,4-dihydro-2H-pyrano[6,5-f]chromen-3-yl]-5-methoxyphenol | 338.43 | 36.21 | 0.52 | *Glycyrrhiza uralensis* Fisch. |
| MOL004980 | Inflacoumarin A | 322.38 | 39.71 | 0.33 | *Glycyrrhiza uralensis* Fisch. |
| MOL004985 | icos-5-enoic acid | 310.58 | 30.7 | 0.2 | *Glycyrrhiza uralensis* Fisch. |
| MOL004988 | Kanzonol F | 420.54 | 32.47 | 0.89 | *Glycyrrhiza uralensis* Fisch. |
| MOL004989 | 6-prenylated eriodictyol | 356.4 | 39.22 | 0.41 | *Glycyrrhiza uralensis* Fisch. |
| MOL004990 | 7,2',4'-trihydroxy－5-methoxy-3－arylcoumarin | 300.28 | 83.71 | 0.27 | *Glycyrrhiza uralensis* Fisch. |
| MOL004991 | 7-Acetoxy-2-methylisoflavone | 294.32 | 38.92 | 0.26 | *Glycyrrhiza uralensis* Fisch. |
| MOL004993 | 8-prenylated eriodictyol | 356.4 | 53.79 | 0.4 | *Glycyrrhiza uralensis* Fisch. |
| MOL004996 | gadelaidic acid | 310.58 | 30.7 | 0.2 | *Glycyrrhiza uralensis* Fisch. |
| MOL000500 | Vestitol | 272.32 | 74.66 | 0.21 | *Glycyrrhiza uralensis* Fisch. |
| MOL005000 | Gancaonin G | 352.41 | 60.44 | 0.39 | *Glycyrrhiza uralensis* Fisch. |
| MOL005001 | Gancaonin H | 420.49 | 50.1 | 0.78 | *Glycyrrhiza uralensis* Fisch. |
| MOL005003 | Licoagrocarpin | 338.43 | 58.81 | 0.58 | *Glycyrrhiza uralensis* Fisch. |
| MOL005007 | Glyasperins M | 368.41 | 72.67 | 0.59 | *Glycyrrhiza uralensis* Fisch. |
| MOL005008 | Glycyrrhiza flavonol A | 370.38 | 41.28 | 0.6 | *Glycyrrhiza uralensis* Fisch. |
| MOL005012 | Licoagroisoflavone | 336.36 | 57.28 | 0.49 | *Glycyrrhiza uralensis* Fisch. |
| MOL005013 | 18α-hydroxyglycyrrhetic acid | 486.76 | 41.16 | 0.71 | *Glycyrrhiza uralensis* Fisch. |
| MOL005016 | Odoratin | 314.31 | 49.95 | 0.3 | *Glycyrrhiza uralensis* Fisch. |
| MOL005017 | Phaseol | 336.36 | 78.77 | 0.58 | *Glycyrrhiza uralensis* Fisch. |
| MOL005018 | Xambioona | 388.49 | 54.85 | 0.87 | *Glycyrrhiza uralensis* Fisch. |
| MOL005020 | dehydroglyasperins C | 340.4 | 53.82 | 0.37 | *Glycyrrhiza uralensis* Fisch. |
| MOL000098 | quercetin | 302.25 | 46.43 | 0.28 | *Glycyrrhiza uralensis* Fisch. |
| MOL000359 | sitosterol | 414.79 | 36.91 | 0.75 | *Aconitum carmichaelii* Debx., *Glycyrrhiza uralensis* Fisch., *Zingiber officinale* Rosc. |
| MOL002211 | 11,14-eicosadienoic acid | 308.56 | 39.99 | 0.2 | *Aconitum carmichaelii* Debx. |
| MOL002388 | Delphin_qt | 303.26 | 57.76 | 0.28 | *Aconitum carmichaelii* Debx. |
| MOL002392 | Deltoin | 328.39 | 46.69 | 0.37 | *Aconitum carmichaelii* Debx. |
| MOL002393 | Demethyldelavaine A | 700.91 | 34.52 | 0.18 | *Aconitum carmichaelii* Debx. |
| MOL002394 | Demethyldelavaine B | 700.91 | 34.52 | 0.18 | *Aconitum carmichaelii* Debx. |
| MOL002395 | Deoxyandrographolide | 334.5 | 56.3 | 0.31 | *Aconitum carmichaelii* Debx. |
| MOL002397 | karakoline | 377.58 | 51.73 | 0.73 | *Aconitum carmichaelii* Debx. |
| MOL002398 | Karanjin | 292.3 | 69.56 | 0.34 | *Aconitum carmichaelii* Debx. |
| MOL002401 | Neokadsuranic acid B | 452.74 | 43.1 | 0.85 | *Aconitum carmichaelii* Debx. |
| MOL002406 | 2,7-Dideacetyl-2,7-dibenzoyl-taxayunnanine F | 776.9 | 39.43 | 0.38 | *Aconitum carmichaelii* Debx. |
| MOL002410 | benzoylnapelline | 463.67 | 34.06 | 0.53 | *Aconitum carmichaelii* Debx. |
| MOL002415 | 6-Demethyldesoline | 453.64 | 51.87 | 0.66 | *Aconitum carmichaelii* Debx. |
| MOL002416 | deoxyaconitine | 629.82 | 30.96 | 0.24 | *Aconitum carmichaelii* Debx. |
| MOL002419 | (R)-Norcoclaurine | 271.34 | 82.54 | 0.21 | *Aconitum carmichaelii* Debx. |
| MOL002421 | ignavine | 449.59 | 84.08 | 0.25 | *Aconitum carmichaelii* Debx. |
| MOL002422 | isotalatizidine | 407.61 | 50.82 | 0.73 | *Aconitum carmichaelii* Debx. |
| MOL002423 | jesaconitine | 675.85 | 33.41 | 0.19 | *Aconitum carmichaelii* Debx. |
| MOL002433 | (3R,8S,9R,10R,13R,14S,17R)-3-hydroxy-4,4,9,13,14-pentamethyl-17-[(E,2R)-6-methyl-7-[(2R,3R,4S,5S,6R)-3,4,5-trihydroxy-6-[[(2R,3R,4S,5S,6R)-3,4,5-trihydroxy-6-(hydroxymethyl)oxan-2-yl]oxymethyl]oxan-2-yl]oxyhept-5-en-2-yl]-1,2,3,7,8,10,12,15,16,17-decahydr | 781.1 | 41.52 | 0.22 | *Aconitum carmichaelii* Debx. |
| MOL002434 | Carnosifloside I_qt | 456.78 | 38.16 | 0.8 | *Aconitum carmichaelii* Debx. |
| MOL000538 | hypaconitine | 615.79 | 31.39 | 0.26 | *Aconitum carmichaelii* Debx. |
| MOL003896 | 7-Methoxy-2-methyl isoflavone | 266.31 | 42.56 | 0.2 | *Codonopsis pilosula* (Franch.) Nannf., *Glycyrrhiza uralensis* Fisch. |
| MOL007059 | 3-beta-Hydroxymethyllenetanshiquinone | 294.32 | 32.16 | 0.41 | *Codonopsis pilosula* (Franch.) Nannf. |
| MOL008391 | 5alpha-Stigmastan-3,6-dione | 428.77 | 33.12 | 0.79 | *Codonopsis pilosula* (Franch.) Nannf. |
| MOL000006 | luteolin | 286.25 | 36.16 | 0.25 | *Codonopsis pilosula* (Franch.) Nannf. |
| MOL006774 | stigmast-7-enol | 414.79 | 37.42 | 0.75 | *Codonopsis pilosula* (Franch.) Nannf. |
| MOL008393 | 7-(beta-Xylosyl)cephalomannine_qt | 830.02 | 38.33 | 0.29 | *Codonopsis pilosula* (Franch.) Nannf. |
| MOL006554 | Taraxerol | 426.8 | 38.4 | 0.77 | *Codonopsis pilosula* (Franch.) Nannf. |
| MOL004492 | Chrysanthemaxanthin | 584.96 | 38.72 | 0.58 | *Codonopsis pilosula* (Franch.) Nannf. |
| MOL007514 | methyl icosa-11,14-dienoate | 322.59 | 39.67 | 0.23 | *Codonopsis pilosula* (Franch.) Nannf. |
| MOL008406 | Spinoside A | 716.95 | 39.97 | 0.4 | *Codonopsis pilosula* (Franch.) Nannf. |
| MOL008411 | 11-Hydroxyrankinidine | 356.46 | 40 | 0.66 | *Codonopsis pilosula* (Franch.) Nannf. |
| MOL001006 | poriferasta-7,22E-dien-3beta-ol | 412.77 | 42.98 | 0.76 | *Codonopsis pilosula* (Franch.) Nannf. |
| MOL004355 | Spinasterol | 412.77 | 42.98 | 0.76 | *Codonopsis pilosula* (Franch.) Nannf. |
| MOL002879 | Diop | 390.62 | 43.59 | 0.39 | *Codonopsis pilosula* (Franch.) Nannf. |
| MOL003036 | ZINC03978781 | 412.77 | 43.83 | 0.76 | *Codonopsis pilosula* (Franch.) Nannf. |
| MOL000449 | Stigmasterol | 412.77 | 43.83 | 0.76 | *Codonopsis pilosula* (Franch.) Nannf. |
| MOL008407 | (8S,9S,10R,13R,14S,17R)-17-[(E,2R,5S)-5-ethyl-6-methylhept-3-en-2-yl]-10,13-dimethyl-1,2,4,7,8,9,11,12,14,15,16,17-dodecahydrocyclopenta[a]phenanthren-3-one | 410.75 | 45.4 | 0.76 | *Codonopsis pilosula* (Franch.) Nannf. |
| MOL008397 | Daturilin | 436.64 | 50.37 | 0.77 | *Codonopsis pilosula* (Franch.) Nannf. |
| MOL008400 | glycitein | 284.28 | 50.48 | 0.24 | *Codonopsis pilosula* (Franch.) Nannf. |
| MOL005321 | Frutinone A | 264.24 | 65.9 | 0.34 | *Codonopsis pilosula* (Franch.) Nannf. |
| MOL002140 | Perlolyrine | 264.3 | 65.95 | 0.27 | *Codonopsis pilosula* (Franch.) Nannf. |
| MOL000020 | 12-senecioyl-2E,8E,10E-atractylentriol | 312.39 | 62.4 | 0.22 | *Atractylodes macrocephala* Koidz. |
| MOL000021 | 14-acetyl-12-senecioyl-2E,8E,10E-atractylentriol | 355.44 | 60.31 | 0.31 | *Atractylodes macrocephala* Koidz. |
| MOL000022 | 14-acetyl-12-senecioyl-2E,8Z,10E-atractylentriol | 356.45 | 63.37 | 0.3 | *Atractylodes macrocephala* Koidz. |
| MOL000028 | α-Amyrin | 426.8 | 39.51 | 0.76 | *Atractylodes macrocephala* Koidz. |
| MOL000033 | (3S,8S,9S,10R,13R,14S,17R)-10,13-dimethyl-17-[(2R,5S)-5-propan-2-yloctan-2-yl]-2,3,4,7,8,9,11,12,14,15,16,17-dodecahydro-1H-cyclopenta[a]phenanthren-3-ol | 428.82 | 36.23 | 0.78 | *Atractylodes macrocephala* Koidz. |
| MOL000049 | 3β-acetoxyatractylone | 274.39 | 54.07 | 0.22 | *Atractylodes macrocephala* Koidz. |
| MOL000072 | 8β-ethoxy atractylenolide Ⅲ | 276.41 | 35.95 | 0.21 | *Atractylodes macrocephala* Koidz. |

Table S6 Functions of of therapeutic target genes based on KEGG pathway analysis

| ID | Term | Count | Genes | FDR |
| --- | --- | --- | --- | --- |
| hsa05200 | Pathways in cancer | 20 | *CDKN1A, TGFB1, NOS2, STAT1, SLC2A1, CXCR4, BRAF, KLK3, FASLG, PRKCA, PTGS2, EGFR, VEGFA, IL6, EDNRB, CDK6, PIK3CA, KIT, ERBB2, RAF1* | 1.13E-08 |
| hsa04151 | PI3K-Akt signaling pathway | 17 | *CDKN1A, SYK, FASLG, PRKCA, EGFR, IL2, VEGFA, IL4, COL1A1, IL6, CDK6, PIK3CA, KIT, SPP1, KDR, RAF1, TLR4* | 2.91E-07 |
| hsa05205 | Proteoglycans in cancer | 15 | *CDKN1A, TGFB1, BRAF, FASLG, PRKCA, MAPK14, TNF, EGFR, VEGFA, PIK3CA, ERBB2, KDR, EZR, RAF1, TLR4* | 2.43E-08 |
| hsa05142 | Chagas disease (American trypanosomiasis) | 13 | *IL10, TGFB1, ACE, NOS2, FASLG, MAPK14, TNF, IL2, IL6, IFNG, PIK3CA, IL1B, TLR4* | 3.87E-09 |
| hsa05164 | Influenza A | 13 | *STAT1, EIF2AK2, FASLG, PRKCA, MAPK14, TNF, IL1A, IL6, IFNG, PIK3CA, IL1B, RAF1, TLR4* | 2.91E-07 |
| hsa05152 | Tuberculosis | 13 | *IL10, TGFB1, SYK, NOS2, STAT1, MAPK14, TNF, IL1A, IL6, IFNG, IL1B, RAF1, TLR4* | 3.09E-07 |
| hsa05140 | Leishmaniasis | 12 | *IL10, IL4, IL1A, TGFB1, IFNG, NOS2, STAT1, IL1B, MAPK14, PTGS2, TNF, TLR4* | 1.94E-09 |
| hsa04066 | HIF-1 signaling pathway | 12 | *IL6, CDKN1A, IFNG, PIK3CA, NOS2, ERBB2, NPPA, SLC2A1, PRKCA, TLR4, EGFR, VEGFA* | 1.13E-08 |
| hsa05162 | Measles | 12 | *IL4, IL1A, IL6, CDK6, IFNG, PIK3CA, STAT1, IL1B, EIF2AK2, FASLG, TLR4, IL2* | 2.35E-07 |
| hsa04060 | Cytokine-cytokine receptor interaction | 12 | *IL10, IL4, IL1A, IL6, TGFB1, IFNG, IL1B, CXCR4, FASLG, CCR5, TNF, IL2* | 3.42E-05 |
| hsa05321 | Inflammatory bowel disease (IBD) | 11 | *IL10, IL4, IL1A, IL6, TGFB1, IFNG, STAT1, IL1B, TNF, TLR4, IL2* | 5.34E-09 |
| hsa05146 | Amoebiasis | 11 | *IL10, COL1A1, IL6, TGFB1, IFNG, PIK3CA, NOS2, IL1B, PRKCA, TNF, TLR4* | 2.81E-07 |
| hsa05161 | Hepatitis B | 11 | *IL6, CDKN1A, TGFB1, CDK6, PIK3CA, STAT1, FASLG, PRKCA, RAF1, TNF, TLR4* | 3.35E-06 |
| hsa04380 | Osteoclast differentiation | 10 | *IL1A, TGFB1, IFNG, SYK, PIK3CA, STAT1, IL1B, BTK, MAPK14, TNF* | 1.11E-05 |
| hsa04068 | FoxO signaling pathway | 10 | *IL10, IL6, CDKN1A, TGFB1, PIK3CA, BRAF, FASLG, RAF1, MAPK14, EGFR* | 1.25E-05 |
| hsa04510 | Focal adhesion | 10 | *COL1A1, PIK3CA, ERBB2, SPP1, KDR, BRAF, PRKCA, RAF1, EGFR, VEGFA* | 2.23E-04 |
| hsa04010 | MAPK signaling pathway | 10 | *IL1A, TGFB1, IL1B, BRAF, FASLG, PRKCA, RAF1, MAPK14, TNF, EGFR* | 9.08E-04 |
| hsa05206 | MicroRNAs in cancer | 10 | *CDKN1A, ABCB1, CDK6, ERBB2, PRKCA, EZR, RAF1, PTGS2, EGFR, VEGFA* | 0.002022668 |
| hsa05212 | Pancreatic cancer | 9 | *TGFB1, CDK6, PIK3CA, STAT1, ERBB2, BRAF, RAF1, EGFR, VEGFA* | 6.86E-07 |
| hsa05145 | Toxoplasmosis | 9 | *IL10, TGFB1, IFNG, NOS2, STAT1, CCR5, MAPK14, TNF, TLR4* | 2.31E-05 |
| hsa04650 | Natural killer cell mediated cytotoxicity | 9 | *IFNG, SYK, PIK3CA, PRF1, BRAF, FASLG, PRKCA, RAF1, TNF* | 4.41E-05 |
| hsa05160 | Hepatitis C | 9 | *CDKN1A, PIK3CA, STAT1, EIF2AK2, BRAF, RAF1, MAPK14, TNF, EGFR* | 7.21E-05 |
| hsa04015 | Rap1 signaling pathway | 9 | *PIK3CA, KIT, KDR, BRAF, PRKCA, RAF1, MAPK14, EGFR, VEGFA* | 0.001180907 |
| hsa05332 | Graft-versus-host disease | 8 | *IL1A, IL6, IFNG, IL1B, PRF1, FASLG, TNF, IL2* | 1.70E-07 |
| hsa04664 | Fc epsilon RI signaling pathway | 8 | *IL4, SYK, PIK3CA, BTK, PRKCA, RAF1, MAPK14, TNF* | 1.11E-05 |
| hsa05133 | Pertussis | 8 | *IL10, IL1A, IL6, NOS2, IL1B, MAPK14, TNF, TLR4* | 1.97E-05 |
| hsa05323 | Rheumatoid arthritis | 8 | *IL1A, IL6, TGFB1, IFNG, IL1B, TNF, TLR4, VEGFA* | 4.47E-05 |
| hsa04660 | T cell receptor signaling pathway | 8 | *IL10, IL4, IFNG, PIK3CA, RAF1, MAPK14, TNF, IL2* | 8.76E-05 |
| hsa04620 | Toll-like receptor signaling pathway | 8 | *IL6, PIK3CA, STAT1, IL1B, SPP1, MAPK14, TNF, TLR4* | 1.20E-04 |
| hsa04932 | Non-alcoholic fatty liver disease (NAFLD) | 8 | *IL1A, IL6, TGFB1, PIK3CA, IL1B, ADIPOQ, FASLG, TNF* | 9.08E-04 |
| hsa04014 | Ras signaling pathway | 8 | *PIK3CA, KIT, KDR, FASLG, PRKCA, RAF1, EGFR, VEGFA* | 0.007258688 |
| hsa05166 | HTLV-I infection | 8 | *IL6, CDKN1A, TGFB1, TERT, PIK3CA, SLC2A1, TNF, IL2* | 0.012989341 |
| hsa05143 | African trypanosomiasis | 7 | *IL10, IL6, IFNG, IL1B, FASLG, PRKCA, TNF* | 2.73E-06 |
| hsa05330 | Allograft rejection | 7 | *IL10, IL4, IFNG, PRF1, FASLG, TNF, IL2* | 4.88E-06 |
| hsa04940 | Type I diabetes mellitus | 7 | *IL1A, IFNG, IL1B, PRF1, FASLG, TNF, IL2* | 1.00E-05 |
| hsa05144 | Malaria | 7 | *IL10, IL6, TGFB1, IFNG, IL1B, TNF, TLR4* | 1.97E-05 |
| hsa05223 | Non-small cell lung cancer | 7 | *CDK6, PIK3CA, ERBB2, BRAF, PRKCA, RAF1, EGFR* | 3.83E-05 |
| hsa04370 | VEGF signaling pathway | 7 | *PIK3CA, KDR, PRKCA, RAF1, MAPK14, PTGS2, VEGFA* | 5.65E-05 |
| hsa05230 | Central carbon metabolism in cancer | 7 | *G6PD, PIK3CA, ERBB2, KIT, SLC2A1, RAF1, EGFR* | 7.21E-05 |
| hsa05214 | Glioma | 7 | *CDKN1A, CDK6, PIK3CA, BRAF, PRKCA, RAF1, EGFR* | 7.39E-05 |
| hsa05132 | Salmonella infection | 7 | *IL1A, IL6, IFNG, NOS2, IL1B, MAPK14, TLR4* | 2.50E-04 |
| hsa04012 | ErbB signaling pathway | 7 | *CDKN1A, PIK3CA, ERBB2, BRAF, PRKCA, RAF1, EGFR* | 3.17E-04 |
| hsa05215 | Prostate cancer | 7 | *CDKN1A, PIK3CA, ERBB2, BRAF, KLK3, RAF1, EGFR* | 3.29E-04 |
| hsa04630 | Jak-STAT signaling pathway | 7 | *IL10, IL4, IL6, IFNG, PIK3CA, STAT1, IL2* | 0.003648806 |
| hsa05168 | Herpes simplex infection | 7 | *IL6, IFNG, STAT1, IL1B, EIF2AK2, FASLG, TNF* | 0.010399049 |
| hsa05219 | Bladder cancer | 6 | *CDKN1A, ERBB2, BRAF, RAF1, EGFR, VEGFA* | 9.08E-05 |
| hsa04672 | Intestinal immune network for IgA production | 6 | *IL10, IL4, IL6, TGFB1, CXCR4, IL2* | 1.68E-04 |
| hsa05211 | Renal cell carcinoma | 6 | *TGFB1, PIK3CA, SLC2A1, BRAF, RAF1, VEGFA* | 7.54E-04 |
| hsa05218 | Melanoma | 6 | *CDKN1A, CDK6, PIK3CA, BRAF, RAF1, EGFR* | 9.87E-04 |
| hsa05220 | Chronic myeloid leukemia | 6 | *CDKN1A, TGFB1, CDK6, PIK3CA, BRAF, RAF1* | 0.001029793 |
| hsa04064 | NF-kappa B signaling pathway | 6 | *SYK, IL1B, BTK, PTGS2, TNF, TLR4* | 0.002252222 |
| hsa04640 | Hematopoietic cell lineage | 6 | *IL4, IL1A, IL6, IL1B, KIT, TNF* | 0.002252222 |
| hsa04668 | TNF signaling pathway | 6 | *IL6, PIK3CA, IL1B, MAPK14, PTGS2, TNF* | 0.005225349 |
| hsa04726 | Serotonergic synapse | 6 | *MAOA, HTR1A, BRAF, PRKCA, RAF1, PTGS2* | 0.006016176 |
| hsa04062 | Chemokine signaling pathway | 6 | *PIK3CA, STAT1, CXCR4, BRAF, CCR5, RAF1* | 0.037213766 |
| hsa05203 | Viral carcinogenesis | 6 | *CDKN1A, CDK6, SYK, PIK3CA, EIF2AK2, CCR5* | 0.048778759 |
| hsa05320 | Autoimmune thyroid disease | 5 | *IL10, IL4, PRF1, FASLG, IL2* | 0.002425516 |
| hsa05213 | Endometrial cancer | 5 | *PIK3CA, ERBB2, BRAF, RAF1, EGFR* | 0.002425516 |
| hsa04976 | Bile secretion | 5 | *ABCB1, SLC2A1, NR1H4, CFTR, ABCG2* | 0.006411527 |
| hsa04919 | Thyroid hormone signaling pathway | 5 | *PIK3CA, STAT1, SLC2A1, PRKCA, RAF1* | 0.031516955 |
| hsa04670 | Leukocyte transendothelial migration | 5 | *PIK3CA, CXCR4, PRKCA, EZR, MAPK14* | 0.031516955 |
| hsa04071 | Sphingolipid signaling pathway | 5 | *PIK3CA, PRKCA, RAF1, MAPK14, TNF* | 0.035167295 |
| hsa04722 | Neurotrophin signaling pathway | 5 | *PIK3CA, BRAF, FASLG, RAF1, MAPK14* | 0.035167295 |
| hsa05169 | Epstein-Barr virus infection | 5 | *CDKN1A, SYK, PIK3CA, EIF2AK2, MAPK14* | 0.036040455 |
| hsa04611 | Platelet activation | 5 | *COL1A1, SYK, PIK3CA, BTK, MAPK14* | 0.041257256 |
| hsa04960 | Aldosterone-regulated sodium reabsorption | 4 | *PIK3CA, PRKCA, KCNJ1, NR3C2* | 0.009492466 |
| hsa05134 | Legionellosis | 4 | *IL6, IL1B, TNF, TLR4* | 0.0222626 |
| hsa04621 | NOD-like receptor signaling pathway | 4 | *IL6, IL1B, MAPK14, TNF* | 0.023765715 |
| hsa05221 | Acute myeloid leukemia | 4 | *PIK3CA, KIT, BRAF, RAF1* | 0.023765715 |
| hsa04150 | mTOR signaling pathway | 4 | *PIK3CA, BRAF, PRKCA, TNF* | 0.025705179 |
| hsa05210 | Colorectal cancer | 4 | *TGFB1, PIK3CA, BRAF, RAF1* | 0.030243763 |
| hsa00982 | Drug metabolism - cytochrome P450 | 4 | *GSTM1, MAOA, GSTA1, CYP3A4* | 0.035770518 |
| hsa04662 | B cell receptor signaling pathway | 4 | *SYK, PIK3CA, BTK, RAF1* | 0.03610316 |
| hsa04917 | Prolactin signaling pathway | 4 | *PIK3CA, STAT1, RAF1, MAPK14* | 0.037817637 |
| hsa04971 | Gastric acid secretion | 4 | *PRKCA, EZR, KCNJ1, CFTR* | 0.040089185 |
| hsa05410 | Hypertrophic cardiomyopathy (HCM) | 4 | *IL6, TGFB1, ACE, TNF* | 0.045664313 |
| hsa05204 | Chemical carcinogenesis | 4 | *GSTM1, GSTA1, CYP3A4, PTGS2* | 0.048077491 |
| hsa05310 | Asthma | 3 | *IL10, IL4, TNF* | 0.042087135 |

Table S7 Informations of 45 kernel ingredients of FLZP

| MOL ID | Compounds | CAS | OB (%) | DL | Degree |
| --- | --- | --- | --- | --- | --- |
| MOL000098 | quercetin | 117-39-5 | 46.43 | 0.28 | 29 |
| MOL000006 | luteolin | 491-70-3 | 36.16 | 0.25 | 21 |
| MOL004835 | Glypallichalcone | 146763-58-8 | 61.6 | 0.19 | 18 |
| MOL003896 | 7-Methoxy-2-methyl isoflavone | 82517-12-2 | 42.56 | 0.2 | 18 |
| MOL004848 | licochalcone G | / | 49.25 | 0.32 | 17 |
| MOL000422 | kaempferol | 520-18-3 | 41.88 | 0.24 | 17 |
| MOL004974 | 3'-Methoxyglabridin | / | 46.16 | 0.57 | 16 |
| MOL004961 | Quercetin der. | 4382-17-6 | 46.45 | 0.33 | 15 |
| MOL004841 | Licochalcone B | 58749-23-8 | 76.76 | 0.19 | 15 |
| MOL000497 | licochalcone a | 58749-22-7 | 40.79 | 0.29 | 15 |
| MOL000359 | sitosterol | 5779-62-4 | 36.91 | 0.75 | 15 |
| MOL000239 | Jaranol | 3301-49-3 | 50.83 | 0.29 | 15 |
| MOL004991 | 7-Acetoxy-2-methylisoflavone | 3211-63-0 | 38.92 | 0.26 | 14 |
| MOL002514 | Sexangularetin | 571-74-4 | 62.86 | 0.3 | 14 |
| MOL005012 | Licoagroisoflavone | / | 57.28 | 0.49 | 13 |
| MOL004990 | 7,2',4'-trihydroxy－5-methoxy-3－arylcoumarin | 1092952-62-9 | 83.71 | 0.27 | 13 |
| MOL004905 | 3,22-Dihydroxy-11-oxo-delta(12)-oleanene-27-alpha-methoxycarbonyl-29-oic acid | 123914-44-3 | 34.32 | 0.55 | 13 |
| MOL004898 | (E)-3-[3,4-dihydroxy-5-(3-methylbut-2-enyl)phenyl]-1-(2,4-dihydroxyphenyl)prop-2-en-1-one | / | 46.27 | 0.31 | 13 |
| MOL004828 | Glepidotin A | 42193-83-9 | 44.72 | 0.35 | 13 |
| MOL004814 | Isotrifoliol | 329319-08-6 | 31.94 | 0.42 | 13 |
| MOL004966 | 3'-Hydroxy-4'-O-Methylglabridin | / | 43.71 | 0.57 | 12 |
| MOL004908 | Glabridin | 59870-68-7 | 53.25 | 0.47 | 12 |
| MOL004815 | (E)-1-(2,4-dihydroxyphenyl)-3-(2,2-dimethylchromen-6-yl)prop-2-en-1-one | / | 39.62 | 0.35 | 12 |
| MOL002406 | 2,7-Dideacetyl-2,7-dibenzoyl-taxayunnanine F | / | 39.43 | 0.38 | 12 |
| MOL002398 | Karanjin | 521-88-0 | 69.56 | 0.34 | 12 |
| MOL000500 | Vestitol | 20879-05-4 | 74.66 | 0.21 | 12 |
| MOL000392 | formononetin | 485-72-3 | 69.67 | 0.21 | 12 |
| MOL000354 | isorhamnetin | 480-19-3 | 49.6 | 0.31 | 12 |
| MOL008400 | glycitein | 40957-83-3 | 50.48 | 0.24 | 11 |
| MOL008393 | 7-(beta-Xylosyl)cephalomannine_qt | / | 38.33 | 0.29 | 11 |
| MOL005016 | Odoratin | 53948-00-8 | 49.95 | 0.3 | 11 |
| MOL005007 | Glyasperins M | / | 72.67 | 0.59 | 11 |
| MOL004957 | isoformononetin | 486-63-5 | 38.37 | 0.21 | 11 |
| MOL004949 | Isolicoflavonol | 94805-83-1 | 45.17 | 0.42 | 11 |
| MOL004810 | glyasperin F | 145382-61-2 | 75.84 | 0.54 | 11 |
| MOL004328 | naringenin | 480-41-1 | 59.29 | 0.21 | 11 |
| MOL004978 | 2-[(3R)-8,8-dimethyl-3,4-dihydro-2H-pyrano[6,5-f]chromen-3-yl]-5-methoxyphenol | / | 36.21 | 0.52 | 10 |
| MOL004911 | Glabrene | 60008-03-9 | 46.27 | 0.44 | 10 |
| MOL004910 | Glabranin | 41983-91-9 | 52.9 | 0.31 | 10 |
| MOL004879 | Glycyrin | 66056-18-6 | 52.61 | 0.47 | 10 |
| MOL004808 | glyasperin B | 142488-54-8 | 65.22 | 0.44 | 10 |
| MOL002565 | Medicarpin | 32383-76-9 | 49.22 | 0.34 | 10 |
| MOL002395 | Deoxyandrographolide | 79233-15-1 | 56.3 | 0.31 | 10 |
| MOL002392 | Deltoin | 19662-71-6 | 46.69 | 0.37 | 10 |
| MOL000417 | Calycosin | 20575-57-9 | 47.75 | 0.24 | 10 |
